# Dual role for CXCL12 signaling in semilunar valve development

**DOI:** 10.1016/j.celrep.2021.109610

**Published:** 2021-08-24

**Authors:** Liam A. Ridge, Dania Kewbank, Dagmar Schütz, Ralf Stumm, Peter J. Scambler, Sarah Ivins

**Affiliations:** 1 Developmental Biology of Birth Defects, UCL Great Ormond Street Institute of Child Health, 30 Guilford Street, London WC1N 1EH, UK; 2 Institute of Pharmacology and Toxicology, Jena University Hospital, Friedrich Schiller University Jena, Jena 07747, Germany

**Keywords:** cell migration, CXCL12, CXCR4, CXCR7, outflow tract, semilunar valves, valve dysplasia, valve hyperplasia, valve primordia

## Abstract

*Cxcl12*-null embryos have dysplastic, misaligned, and hyperplastic semilunar valves (SLVs). In this study, we show that CXCL12 signaling via its receptor CXCR4 fulfills distinct roles at different stages of SLV development, acting initially as a guidance cue to pattern cellular distribution within the valve primordia during the endocardial-to-mesenchymal transition (endoMT) phase and later regulating mesenchymal cell proliferation during SLV remodeling. Transient, anteriorly localized puncta of internalized CXCR4 are observed in cells undergoing endoMT. *In vitro*, CXCR4^+^ cell orientation in response to CXCL12 requires phosphatidylinositol 3-kinase (PI3K) signaling and is inhibited by suppression of endocytosis. This dynamic intracellular localization of CXCR4 during SLV development is related to CXCL12 availability, potentially enabling activation of divergent downstream signaling pathways at key developmental stages. Importantly, *Cxcr7^-/-^* mutants display evidence of excessive CXCL12 signaling, indicating a likely role for atypical chemokine receptor CXCR7 in regulating ligand bioavailability and thus CXCR4 signaling output during SLV morphogenesis.

## Highlights

• Semilunar valves are dysplastic, misaligned, and hyperplastic in *Cxcl12*-null mice
• Cells undergoing endoMT express punctate CXCR4 and require CXCL12 for guidance
• CXCL12 regulates proliferation of valve mesenchymal cells after E12.5
• CXCR7 is required as a sink for CXCL12 during valve development

## Graphical abstract

**Figure:**
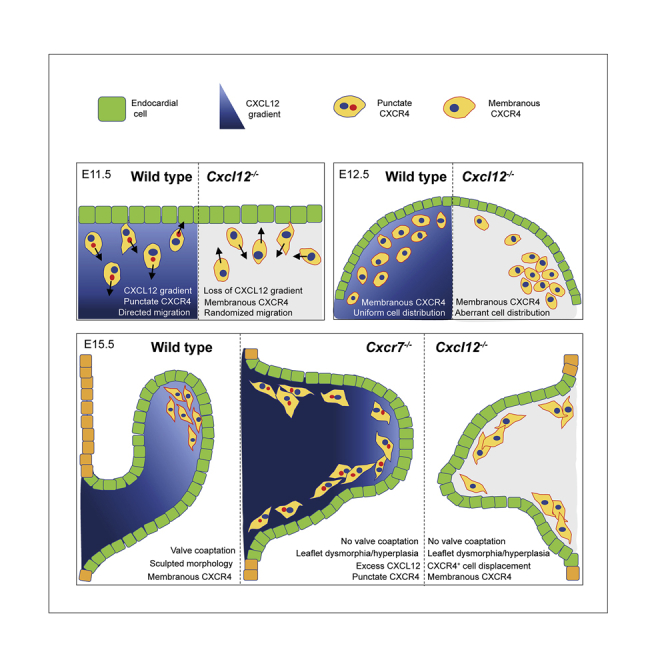

## Teaser

Using *in vivo* and *in vitro* analysis, Ridge et al. show that the CXCL12/CXCR4/CXCR7 signaling axis fulfills distinct roles at different stages of mouse semilunar valve development. Regulation of ligand availability and CXCR4 activity by CXCR7 may be key to enabling alternative functional outcomes from chemokine signaling within a single tissue.

## Introduction

The semilunar valves (SLVs) are positioned at the base of the great arteries and ensure unidirectional blood flow (O’Donnell and Yutzey, 2020; Spicer et al., 2014). The SLVs develop from the endocardial cushions, regions of cardiac jelly located between the endothelium and myocardium of the outflow tract (OFT) and consisting of the main cushions (superior and inferior) and smaller, intercalated cushions (Phillips et al., 2013; Sizarov et al., 2012). Valvulogenesis is initiated by endothelial-to-mesenchymal transition (endoMT) (Chakraborty et al., 2010; Männer and Yelbuz, 2019; Miquerol and Kelly, 2013; O’Donnell and Yutzey, 2020), leading to invasion of the endocardial cushions by mesenchymal precursors originating from the overlying endocardium, between embryonic day 10.5 (E10.5) and E11.5. Subsequently, fusion of the main cushions and septation of the OFT give rise to the tricuspid SLVs by E12.5 (Combs and Yutzey, 2009; Lin et al., 2012). Neural crest (NC) also contributes substantially to the main OFT cushions (de Lange et al., 2004; Liu et al., 2018; Nakamura et al., 2006; Phillips et al., 2013), although the intercalated cushions have been shown to originate largely from second heart field (SHF) (Eley et al., 2018; Sizarov et al., 2012). Perfect coaptation of valve leaflets is essential to avoid backflow of blood into the heart. Sculpting and specification of valve primordia into mature leaflets with stratified extracellular matrix (ECM) is directed by both proliferation and differentiation of mesenchymal cells (Combs and Yutzey, 2009; Lin et al., 2012; MacGrogan et al., 2014; O’Donnell and Yutzey, 2020) and regulated, at least in part, by hemodynamic forces (Goddard et al., 2017; Hsu et al., 2019).

Heart valve defects, including bicuspid aortic valve (BAV), represent the most prevalent class of congenital heart malformations, affecting 1%–5% newborns (Hoffman and Kaplan, 2002; Howard et al., 2019; Lupo et al., 2019). Moreover, heart valve disease is becoming more common among an aging global population (Rostagno, 2019). We previously reported the presence of abnormal SLVs in *Cxcl12-*ablated mouse embryos (Ivins et al., 2015). The chemoattractant cytokine ligand CXCL12 and its reciprocal G protein-coupled receptor (GPCR), CXCR4, are known to guide the migration of numerous cell types during development and adult life (Lewellis and Knaut, 2012). As well as cardiac development (Ivins et al., 2015; Kim et al., 2017; Page et al., 2018), CXCL12 signaling is involved in multiple physiological processes, including angiogenesis, hematopoiesis, neurogenesis, and tumor progression, via effects on chemotaxis, proliferation, and survival (reviewed by Döring et al., 2014; Guyon, 2014; Karpova and Bonig, 2015; Kiefer and Siekmann, 2011; Teicher and Fricker, 2010). Binding of ligand to receptor induces CXCR4 coupling to members of the G_i_ family of proteins, with subsequent downstream activation of divergent intracellular signaling cascades, including phosphatidylinositol 3-kinase (PI3K)/AKT and mitogen-activated protein kinase (MAPK); this is followed by rapid phosphorylation of CXCR4 at its C terminus (by GPCR-specific kinases) and β-arrestin-mediated internalization (Busillo and Benovic, 2007; Luo et al., 2017; Mueller et al., 2013). As part of this desensitization process, internalized CXCR4 is dephosphorylated and can be degraded or recycled to the cell surface (Busillo and Benovic, 2007). In addition, recruitment of β-arrestin may promote MAPK signaling alongside receptor endocytosis (Cheng et al., 2000; Lefkowitz and Shenoy, 2005; Orsini et al., 1999). A second CXCL12 receptor, CXCR7 (ACKR3), has also been implicated in the development of SLVs (Sierro et al., 2007; Yu et al., 2011). CXCR7 can function as a “decoy” receptor in various contexts, sequestering CXCL12 and reducing its bioavailability in order to fine-tune CXCR4-mediated CXCL12 signaling (Abe et al., 2014; Boldajipour et al., 2008; Memi et al., 2013; Naumann et al., 2010; Venkiteswaran et al., 2013).

Although CXCL12, CXCR4, and CXCR7 are all known to be involved in SLV formation, mechanistic details remain unclear. Here, we demonstrate that CXCL12 signaling affects distinct aspects of SLV morphogenesis, with an early requirement for CXCL12 signaling in regulating leaflet shape and alignment and a later role in downregulating the proliferation of valve mesenchymal cells (MCs). Analysis of mutant mouse embryos showed that CXCL12 provides a directional cue for CXCR4^+^ cells undergoing endoMT, a phase associated with the presence of punctate, internalized CXCR4. Furthermore, the ability of CXCR4^+^ OFT MCs to respond to CXCL12 *in vitro* depends on both PI3K signaling and endocytosis. Hyperplasia observed in NC-specific knockouts of CXCR4 indicates the involvement of further cell lineages in SLV remodeling post-endoMT. Finally, analysis of *Cxcr7* null mutants and *ex vivo* receptor inhibition experiments suggest that CXCR7 may act as a chemokine scavenger to modulate CXCL12 levels at different stages of SLV development.

## Results

### *Cxcl12* mutant valves are dysplastic and hyperplastic

**Figure 1:**
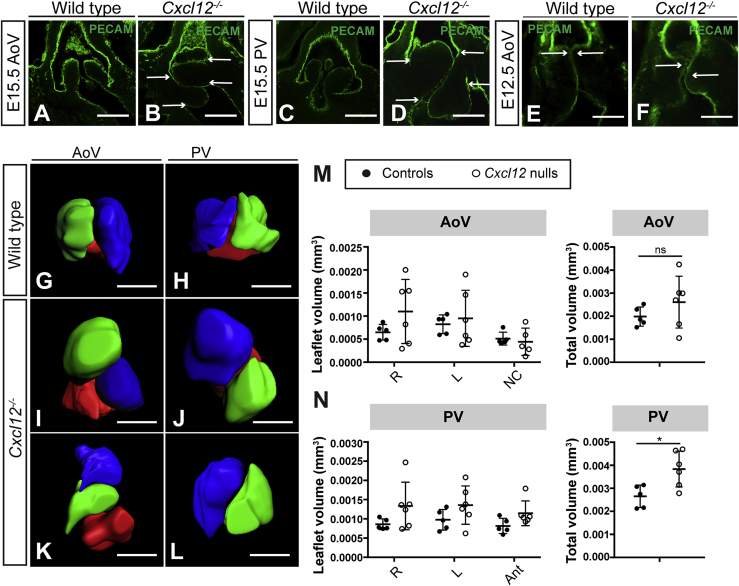
Homozygous mutation of *Cxcl12* causes SLV dysplasia and hyperplasia (A–D) E15.5 wild-type (A and C) and *Cxcl12*^*−/−*^ (B and D) SLVs, whole-mount immunostained with anti-PECAM-1 antibody (confocal images). Arrows in (B) and (D) indicate top and bottom edges of left and right leaflets; leaflet alignment is abnormal in (B) and normal in (D). (E and F) E12.5 wild-type (E) and *Cxcl12*^*−/−*^ (F) AoVs, whole-mount immunostained with anti-PECAM-1 antibody (confocal images). Arrows indicate alignment of the top edges of left and right leaflets, abnormal in (F). (G–L) 3D reconstructions (Imaris) of E15.5 control (G and H) and *Cxcl12*^*−/−*^ SLVs showing leaflet misalignment (I–K), hyperplasia (I, J, and L), and bicuspid PV (L). Leaflet color is as follows: green (right), blue (left), and red (non-coronary or anterior). (M and N) Graphs show individual or combined leaflet volumes (total volume, mm^3^) for AoV (M) and PV (N) in E15.5 control and *Cxcl12*^*−/−*^ hearts. Error bars represent ± SD; ns, non-significant; ^∗^p < 0.05 (unpaired Student’s t test). AoV, aortic valve; PV, pulmonary valve; SLV, semilunar valve. All scale bars represent 100 μm. See also Figure S1.

We previously reported SLV abnormalities in *Cxcl12*-null mouse embryos (*Cxcl12*^*GFP/GFP*^; hereafter referred to as *Cxcl12* null/*Cxcl12*^*−/−*^) at E18.5 (Ivins et al., 2015). Whole-mount and section immunostaining with PECAM-1 antibody confirmed that aortic valve (AoV) and pulmonary valve (PV) leaflets in *Cxcl12* nulls were severely affected at E15.5, with failure of coaptation (Figures 1A–1D). Comparing *Cxcl12*^*−/−*^ (n = 16) to wild-type/*Cxcl12*^*+/−*^ controls (n = 18) at E15.5 revealed dysplasia (defined for the purposes of this study as leaflet malformation with thickening of the hinges) in 100% of null SLVs and misalignment (of left and right leaflets) in 59.4% (19/32 individual SLVs; p < 0.001; with misalignment of either AoV or PV always seen). Dysplasia and subtle misalignment of the valve leaflets was also observed earlier, at E12.5 (n = 11, arrows; Figures 1E and 1F). In addition, histological staining at E18.5 revealed expanded Alcian blue staining (Figures S1A–S1D), suggesting abnormal proteoglycan deposition in *Cxcl12* nulls (n = 3). 3D reconstruction of E15.5 SLVs enabled measurement of leaflet volumes, revealing variable hyperplasia in *Cxcl12* nulls (n = 6; Figures 1G–1N); one case of bicuspid PV was also observed (Figure 1L). Overall, total SLV volume was increased in *Cxcl12* nulls (1.5-fold in the PV and 1.3-fold in the AoV), although this was only significant for the PV (Figures 1M and 1N). However, comparison of mean SLV leaflet area showed a more consistent, significant increase (1.9-fold; see Figure 6F). *Cxcr4* nulls displayed a similar phenotype, with dysplasia, hyperplasia, and misalignment of SLV leaflets observed at E15.5 (n = 6 nulls/controls; Figures S1E–S1H).

### Dynamic expression of CXCR4 and CXCL12 during SLV development

**Figure 2:**
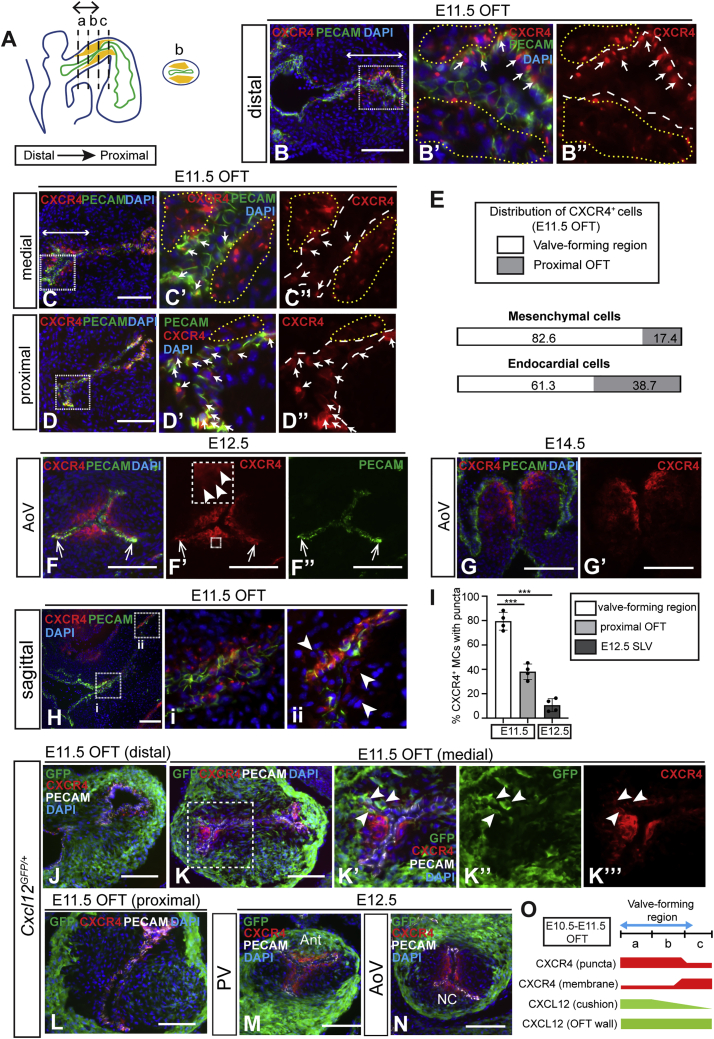
Dynamic expression of CXCR4 and CXCL12 in the OFT and developing SLVs (A) Schematic representation of E10.5–E11.5 heart (sagittal view) showing endocardium (green), endocardial cushions (yellow), and distal-proximal axis of OFT. Sectors (a), (b), and (c) identify distal, medial, and proximal OFT cushion, respectively; transverse section from region (b) (medial OFT) is also depicted. Double-headed arrow indicates valve-forming region. (B–D’’) E11.5 wild-type OFT immunostained with anti-CXCR4 and PECAM-1 antibodies: distal region (a) in (B)–(B”); medial region (b) in (C)–(C”); and proximal region (c) in (D)–(D”). Boxed areas in (B)–(D) are shown enlarged in neighboring panels; double-headed arrows in (B) and (C) indicate valve primordia. Arrows in (B’), (B”), (C’), (C”), (D’), and (D”) show CXCR4^+^ ECs; yellow dashed lines indicate sub-endocardial CXCR4^+^ MCs, and white dashed lines indicate endocardium. (E) Bar graphs show relative distribution of CXCR4^+^ MCs (PECAM-1^−^) and ECs (PECAM-1^+^) within the OFT (valve-forming region and proximal OFT cushion) at E11.5. (F–G’) E12.5 (F–F’’) and E14.5 (G and G’) AoV, immunostained as above. Faint puncta in (F’) are shown enlarged in inset box; arrows in (F)–(F’’) indicate CXCR4^+^ ECs in SLV commissures. (H) Sagittal section of E11.5 wild-type OFT (immunostained as above). Boxed areas showing distal (i) and proximal (ii) OFT are enlarged in neighboring panels. Arrowheads indicate CXCR4^+^ MCs (PECAM-1^−^) in proximal cushion. (I) Graph shows proportion of CXCR4^+^ MCs with punctate CXCR4 in valve-forming and proximal cushion regions of E11.5 OFT (n = 4) and E12.5 SLVs (n = 4) as a percentage of total CXCR4^+^ MCs. Error bars represent ± SD; ^∗∗∗^p ≤ 0.001 (unpaired Student’s t test). (J–N) *Cxcl12*^*GFP/+*^ E11.5 OFT cushion (J–L) and E12.5 SLV (M and N) sections immunostained with anti-GFP, anti-CXCR4, and anti-PECAM-1 antibodies. Boxed region in (K) is shown enlarged in neighboring panels; arrowheads indicate GFP^+^/CXCR4^+^ cells. (O) Schematic showing relative levels of punctate or membranous CXCR4 (red) in MCs and ECs, and CXCL12 in the OFT wall and cushions (green) in distal, medial, and proximal OFT. Ant, anterior; EC, endocardial cell; MC, mesenchymal cell; NC, non-coronary; OFT, outflow tract; RA, right atrium; RV, right ventricle. Scale bars represent 100 μm. See also Figure S2.

In order to understand the basis of the *Cxcl12* null phenotype, we analyzed CXCL12/CXCR4 signaling components in the developing murine OFT and SLVs, revealing a complex and dynamic expression pattern. For our purposes, we considered the OFT cushions divisible into approximately equal distal, medial, and proximal thirds. The site of SLV morphogenesis has been identified as the junction of the distal and proximal OFT cushions (Anderson et al., 2003; Lin et al., 2012; MacGrogan et al., 2014), approximating to the distal and medial cushions (indicated by the double-headed arrow in Figure 2A) and hereinafter termed the valve-forming region. CXCR4 was detected using antibodies specific for the unphosphorylated protein (unphos-CXCR4) (Mueller et al., 2013). At E10.5, CXCR4 was expressed solely in OFT endocardium (distal, medial, and proximal; Figures S2A–S2C”). Endocardial expression was maintained at E11.5, with CXCR4 additionally detected in sub-endocardial MCs (Figures 2B–2D” and 2H–2Hii). Significantly, the localization of CXCR4^+^ MCs correlated closely with the SLV primordia (double-headed arrows in Figures 2B and 2C). CXCR4^+^ MCs were mainly concentrated in the valve-forming region, whereas CXCR4^+^ endocardial cells (ECs) were more evenly distributed along the OFT (Figure 2E). At E12.5, CXCR4 was strongly expressed in sub-endocardially localized MCs, with reduced expression extending further into the leaflet mesenchyme (Figures 2F–2F” and S2G); small numbers of CXCR4^+^ ECs were also observed in the valve commissures (arrows, Figures 2F–2F”). By E14.5, CXCR4 was restricted to mesenchyme and detected mainly at the tips and ventricular (inner) edges of the leaflets (Figures 2G, 2G’, and S2H).

Surprisingly, the subcellular localization of CXCR4 also varied according to both developmental stage and distal-proximal position. At E10.5 and E11.5, single intracellular puncta of CXCR4 (with varying levels of membranous CXCR4) were detected in ECs (E10.5 and E11.5) and MCs (E11.5 only; Figures 2B–2C”, 2Hi, and S2A–S2C”), whereas at E12.5, CXCR4 puncta were only faintly visible in a small number of SLV MCs (arrowheads in inset box, Figure 2F’). A higher frequency of puncta in CXCR4^+^ MCs was observed in the valve-forming region of the OFT (82.2%) compared to proximal OFT (40.1%) and E12.5 leaflets (10.6%) (Figure 2I; see also Figures 2H–2Hii). Notably, the presence of CXCR4 puncta coincided with the developmental window during which endoMT occurs.

*Cxcl12* expression in the OFT was broadly complementary to CXCR4, as determined by GFP fluorescence in *Cxcl12*^*GFP/+*^ hearts. In addition to high expression throughout the OFT wall, CXCL12-GFP was detected at lower levels in the cushions at E10.5–E11.5, declining in a distal-proximal fashion (Figures 2J–2L and S2D–S2F; summarized in Figure 2O). At E11.5, GFP^+^ cushion cells were closely juxtaposed to CXCR4^+^ MCs in the valve-forming region; some double-positive cells were also present (arrowheads, Figures 2K’–2K”’). At E12.5, CXCL12-GFP was mainly confined to the walls of the OFT; however, expression was also observed in leaflets derived from the intercalated cushions, i.e., PV anterior and AoV non-coronary leaflets (Figures 2M and 2N). The proximity of CXCR4^+^ cells to a CXCL12 source within the developing SLV primordia suggests a potential role in shaping and aligning the leaflets.

### CXCR4^+^ mesenchymal cells in the OFT derive from multiple lineages

Cells of endocardial, NC, and SHF origin contribute to the SLVs (Eley et al., 2018; Liu et al., 2018; Nakamura et al., 2006; Phillips et al., 2013). To determine the origin of CXCR4^+^ MCs within the OFT cushions at E11.5, lineage tracing was initially undertaken by crossing *Tie2-Cre* and *ROSA26R*^*YFP/YFP*^ mice to label endocardially derived cells (Figures S2I–S2I”’); lineage^+^ cells were detected using GFP antibody. On average, 62.7% of CXCR4^+^/ PECAM-1^−^ (mesenchymal) cells found in *Tie2-Cre;R26R*^*YFP/+*^ OFT were GFP^+^, showing that they derived from endocardium (n = 3; Figure S2L).

NC cells were analyzed using *Pax3*-*Cre;R26R*^*YFP/+*^ embryos (Figures S2J–S2J”’). We identified two subsets of NC-derived cells within the OFT: mesenchymal “prongs” of CXCR4^−^ cells (asterisks, Figure S2J) located in the central part of the OFT cushions (Anderson et al., 2003) and much smaller numbers of sub-endocardially localized CXCR4^+^ NC-derived cells (arrowheads, Figure S2 J’). The latter CXCR4^+^/PECAM-1^−^/GFP^+^ cells accounted for 13.8% of all CXCR4^+^/PECAM-1^−^ cells in *Pax3*-*Cre;R26R*^*YFP/+*^ OFT (n = 3; Figure S2L). Taking the above data together, approximately 75% of CXCR4^+^ OFT MCs originate from endocardium and NC.

To account for the remainder, we utilized *Mef2c*-*Cre;R26R*^*YFP/+*^ embryos. *Mef2c-AHF-Cre* labels SHF derivatives, including the OFT endocardium (Milgrom-Hoffman et al., 2011; Verzi et al., 2005); however, it also traces a subset of non-endocardially derived MCs that differentiate directly from SHF progenitors in the OFT wall and contribute to the intercalated cushion-derived SLV leaflets (Eley et al., 2018). In *Mef2c*-*Cre;R26R*^*YFP/+*^ OFT, 82% of CXCR4^+^/PECAM-1^−^ cells were also GFP^+^ (n = 3; Figures S2K–S2K”’ and S2L). Thus, approximately 20% of CXCR4^+^ MCs derive directly from non-endocardial SHF progenitors. Overall, these data indicate that, at E11.5, the majority of CXCR4^+^ MCs result from endoMT, with the remainder deriving from SHF and NC. By E12.5, numbers of CXCR4^+^ NC-derived cells were greatly increased, constituting 44.3% and 40.9% of total CXCR4^+^ cells in the AoV and PV respectively (n = 3; Figures S2L–S2P”). This suggested activation of CXCR4 expression in NC cells occurring between E11.5 and E12.5, i.e., after endoMT.

### Loss of *Cxcl12* affects positioning and orientation of cells undergoing endoMT

**Figure 3:**
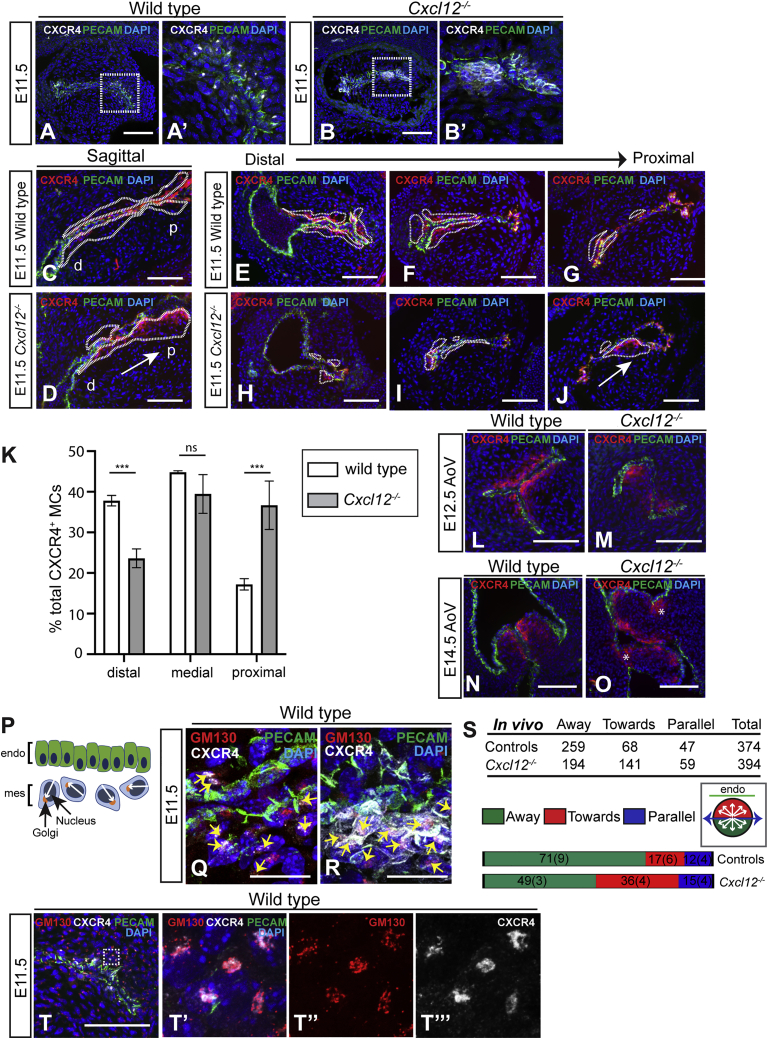
Loss of *Cxcl12* affects positioning and orientation of cells undergoing endoMT (A–B’) E11.5 wild-type (A and A’) and *Cxcl12*^*−/−*^ (B and B’) sections (medial OFT cushion) immunostained with anti-CXCR4 and anti-PECAM-1 antibodies. Boxed regions in (A) and (B) are shown enlarged in (A’) and (B’), respectively. (C and D) Sagittal sections through E11.5 wild-type (C) and *Cxcl12*^*−/−*^ (D) OFT, immunostained as above. Dotted lines delineate CXCR4^+^ MCs. Arrow in (D) indicates displacement of CXCR4^+^ MCs from distal (d) to proximal (p) cushion. (E–J) Transverse sections through E11.5 wild-type (E–G) and *Cxcl12*^*−/−*^ (H–J) distal-proximal OFT, immunostained as above, with CXCR4^+^ MC distribution delineated by dotted lines. Arrow in (J) indicates displacement of CXCR4^+^ MCs toward center of OFT cushion. (K) Distribution of CXCR4^+^ MCs in distal, proximal, and medial OFT as a percentage of total CXCR4^+^ MC number, in wild types (n = 4) and *Cxcl12*^*−/−*^ (n = 5). Error bars represent ± SD; ^∗∗∗^p ≤ 0.001 (unpaired Student’s t test). (L–O) Defective leaflet shape and localization of CXCR4^+^ MCs in E12.5 (M) and E14.5 (O) *Cxcl12*^*−/−*^ AoVs compared to wild types (L) and (N), immunostained as above. Asterisks (O) indicate ectopically localized CXCR4^+^ cells. (P–T) Analysis of CXCR4^+^ MC orientation in E11.5 OFT. See arrows in schematic (P); Golgi position relative to nucleus defines cell anterior. Sections were immunostained with anti-GM130 (Cl.35), anti-CXCR4, and anti-PECAM-1 antibodies (R and S); max z projections of wild-type (Q) and *Cxcl12*^*−/−*^ OFTs (R) are shown, and yellow arrows indicate CXCR4^+^ MC orientation relative to nearest endocardial surface. Graphs in (S) compare cell percentages per orientation (away, toward, or parallel) in *Cxcl12*^*−/−*^ and controls, standard deviation in brackets; table shows cell numbers (p < 0.0001; chi-square = 35.7; 2 degrees of freedom [df]). Inset circular diagram indicates criteria for cell orientation. (T–T’”) Co-localization of CXCR4 puncta with Golgi (detected by anti-GM130 antibody) in E11.5 OFT, co-immunostained with anti-CXCR4 and anti-PECAM-1 antibodies. Boxed region in (T) is shown enlarged in neighboring panels. mes, mesenchyme. Scale bars represent 100 μm except for (Q) and (R), where they represent 20 μm. See also Figure S3.

*Cxcl12* nulls exhibit SLV abnormalities by E12.5, indicating that defects originate during the earliest phase of SLV development. As most CXCR4^+^ MCs in the OFT arise from endoMT, we examined CXCR4 expression at E11.5. While high levels of membranous CXCR4 were observed, CXCR4 puncta were completely absent in *Cxcl12* nulls (Figures 3A–3B’), presumably due to lack of receptor internalization in the absence of CXCL12 (Busillo and Benovic, 2007). Strikingly, the distribution of CXCR4^+^ MCs was disrupted in the valve-forming region of *Cxcl12* nulls, leading to abnormal patterning of the valve primordia (n = 8). We observed displacement of CXCR4^+^ MCs from lateral to central OFT cushion, as well as a significant shift in localization from distal to proximal in *Cxcl12* nulls (Figures 3C–3K). This mis-patterning of the valve primordia resulted in abnormal SLV leaflet morphology and alignment at E12.5 (n = 6 *Cxcl12*^*−/−*^ and controls; Figures 3M and S3B; and see Figure 1F), while at E14.5, CXCR4^+^ MCs were observed in ectopic locations within the dysplastic leaflets, e.g., extending to the hinge and along the aorta-facing surfaces (n = 3; asterisks, Figures 3O and S3D). Furthermore, lineage tracing in *Cxcr4* nulls at E12.5 revealed abnormal accumulations of endocardially derived cells in *Tie2-Cre*;*ROSA26R*^*YFP/+*^;*Cxcr4*^*−/−*^ SLV leaflets (n = 3; Figures S3E–S3F’), as well as perturbed NC-derived cell localization along the ventricular leaflet edges in *Pax3-Cre*;*ROSA26R*^*YFP/+*^;*Cxcr4*^*−/−*^ mutants (n = 3; Figures S3G–S3H’), possibly due to the latter cells being “crowded out” by the abnormally distributed endocardial derivatives. Despite this abnormal cellular distribution, endoMT markers were expressed normally in *Cxcl12* nulls, as shown by immunostaining for SLUG (n = 3; Figures S3I–S3J’) and SOX9 (n = 3; Figures S3K–S3L”). Moreover, the proportions of SLUG^+^ cells in the OFT at E11.5 were similar in nulls and controls (Figure S3M).

Directional cell migration from the endocardium necessitates acquisition of front-back (F-B) polarity in response to external cues. To test whether direction sensing was adversely affected in CXCR4^+^ cells undergoing endoMT in the absence of CXCL12, we examined polarity in E11.5 OFT CXCR4^+^ MCs using the Golgi as an anterior marker (Yadav and Linstedt, 2011). Numbers of cells orientated “away” from, “toward,” or “parallel” with the nearest endocardial surface were quantitated (Figures 3R–3T). The percentage of CXCR4^+^ MCs orientated away from the endocardium was significantly reduced in *Cxcl12* nulls (49.2%) compared to wild-type controls (70.3%, p < 0.0001), suggesting that CXCL12 secreted by the OFT is required for cell guidance during endoMT. Interestingly, co-localization of CXCR4 puncta with the Golgi and a further anterior marker, the centrosome, was observed in both MCs and ECs (Figures 3T–3T”’ and S3N–S3N”’).

### Inhibition of PI3K affects orientation of CXCR4^+^ OFT mesenchymal cells in response to a CXCL12 gradient

**Figure 4:**
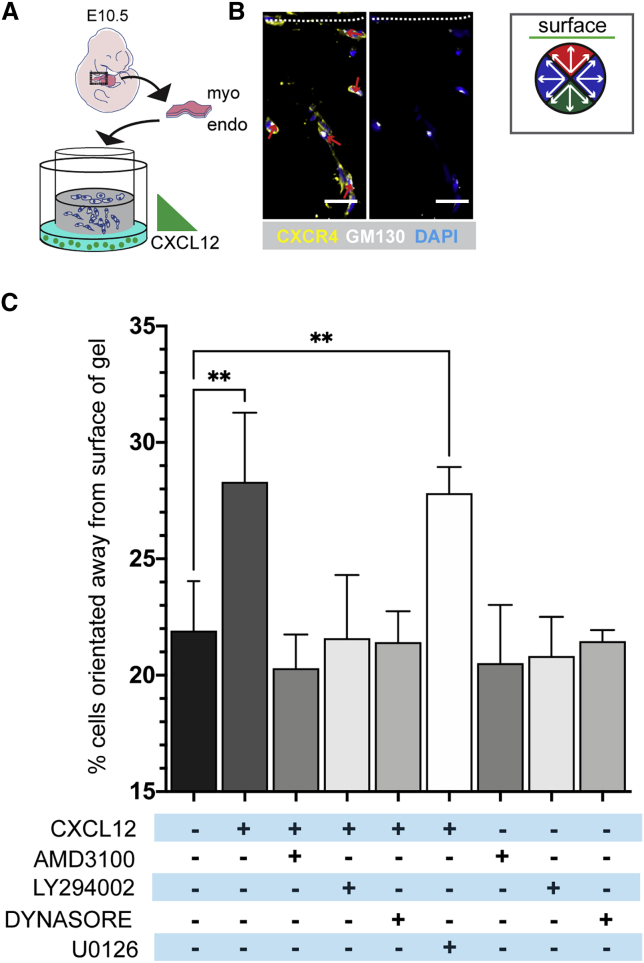
Inhibition of PI3K affects orientation of CXCR4^+^ OFT mesenchymal cells in response to a CXCL12 gradient (A) Schematic representation of OFT explant assay using E10.5 wild-type embryos. (B) Sections of explants in collagen were immunostained with anti-CXCR4 (Cl. 247506) and anti-GM130 (Cl. EP892Y) antibodies; nuclei were stained with DAPI. Arrows indicate cellular orientation in relation to collagen surface (dashed line). Orientations were assigned according to sectors in the circular diagram. (C) Graph shows mean “away” orientated CXCR4^+^ MCs as a percentage of total CXCR4^+^ MCs after indicated treatment (minimum 3 explants per treatment group and >1,400 cells analyzed). Each treatment group mean was compared to the control group (serum-free only, black bar) using a one-way ANOVA test. Bars indicate mean ± SD. Comparisons achieving statistical significance are indicated by asterisks (^∗∗^p ≤ 0.01). endo, endocardium; myo, myocardium. Scale bars represent 25 μm.

We further explored the role of CXCL12/CXCR4 in direction sensing in the OFT using a modified endoMT assay (Di Martino et al., 2017; Figures 4A–4C). Wild-type E10.5 OFTs were explanted onto collagen gels and cultured to allow ingrowth of explant cells (Figure 4A). After explant removal, a CXCL12 gradient (or serum-free medium) was applied, and cellular orientation relation to the gel surface was analyzed, again using the Golgi as an anterior marker (Figure 4B). CXCL12 increased the proportion of CXCR4^+^ cells orientated away from the gel surface (and thus toward the gradient) from 21.9% (in serum-free medium; n = 5) to 28.3% of total cells, i.e., a 1.29-fold increase (n = 10 CXCL12-treated explants; Figure 4C). Inclusion of the CXCR4 inhibitor AMD3100 negated the effect of CXCL12 (n = 4). These results indicate that application of a CXCL12 gradient is sufficient to direct OFT-derived CXCR4^+^ cells away from the gel surface and toward the CXCL12 source, corroborating our *in vivo* observations. The endocytosis inhibitor Dynasore (Macia et al., 2006) also abrogated the effect of CXCL12 (n = 5), showing that receptor internalization was required for direction sensing (Figure 4C).

In order to identify the molecular pathways downstream of CXCL12/CXCR4 required for the orientation response, we treated explants with chemical inhibitors of either the MAPK/ERK (U0126; n = 5) or PI3K/AKT (LY294002; n = 5) signaling cascades prior to application of the CXCL12 gradient. Whereas inhibition of MEK1/2 had no significant effect on the orientation of CXCL12-treated cells, inhibition of PI3K completely abolished the response to CXCL12 (Figure 4C). Treatment with inhibitors alone had no effect on cell orientation. Together, these data suggest that the mechanism by which CXCL12 directs the orientation of migratory CXCR4^+^ MCs is mediated specifically by CXCR4-induced activation of the PI3K/AKT signaling pathway.

### Dysregulated mesenchymal cell proliferation in *Cxcl12* null SLVs

**Figure 5:**
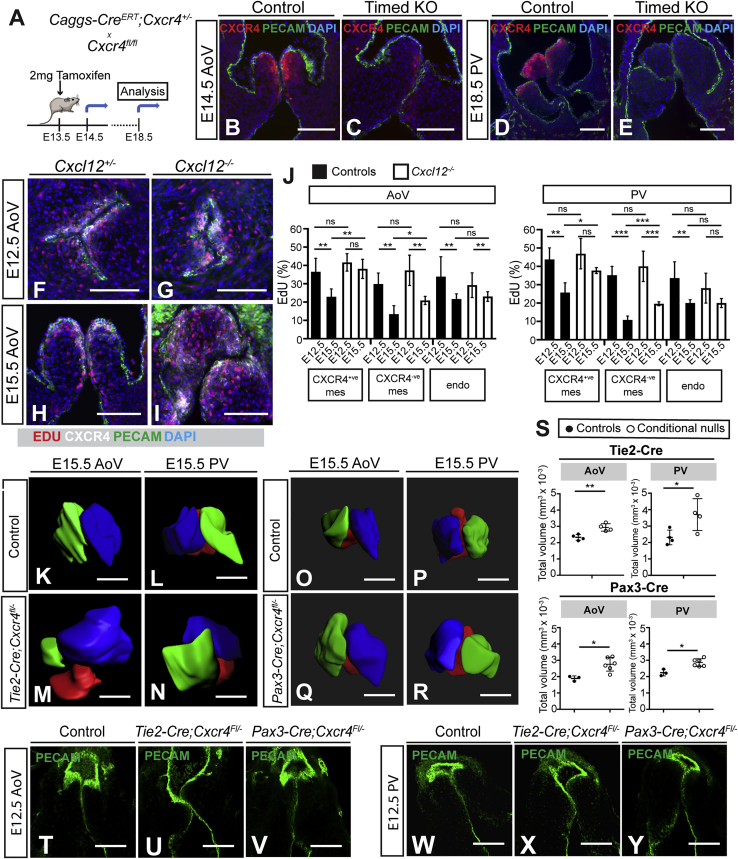
Increased mesenchymal cell proliferation in *Cxcl12*-null SLVs and distinct SLV dysmorphogenesis following tissue-specific mutagenesis of *Cxcr4* (A–E) Timed knockout of CXCR4. Schematic (A) shows breeding scheme to generate *Caggs-Cre*^*ERT*^*;Cxcr4*^*fl/−*^ embryos and timing of tamoxifen injection and analysis. Timed knockout mutant (C and E) and control (*Caggs-Cre*^*ERT*^*;Cxcr4*^*fl/+*^; B and D) SLV sections were immunostained with anti-CXCR4 and anti-PECAM-1 antibodies. (F–J) EdU incorporation in E12.5 and E15.5 SLVs. Wild-type and *Cxcl12*^*−/−*^ AoV sections were immunostained with anti-CXCR4 and anti-PECAM-1 antibodies; red nuclei indicate incorporated EdU (F–I). Graphs in (J) compare EDU^+^ cells (as percentages of total CXCR4^+^ or CXCR4^−^ MCs and total ECs) in *Cxcl12* nulls and controls. Error bars represent ± SD; ^∗^p ≤ 0.05, ^∗∗^p ≤ 0.01, and ^∗∗∗^p ≤ 0.001 (unpaired Student’s t test). (K–R) 3D reconstructions (Imaris) of E15.5 SLVs from controls (K, L, O, and P), *Tie2-Cre; Cxcr4*^*fl/−*^ mutants (M and N), and *Pax3-Cre;Cxcr4*^*fl/−*^ mutants (Q and R). Leaflet color is as follows: green (right); blue (left); and red (non-coronary or anterior). (S) Graphs show total SLV volumes (mm^3^) for E15.5 *Tie2-Cre;Cxcr4*^*fl/−*^ and *Pax3-Cre;Cxcr4*^*fl/−*^ hearts (compared to *Cxcr4*^*fl/−*^ littermate controls. Error bars represent ± SD; ^∗^p < 0.05 and ^∗∗^p < 0.01 (unpaired Student’s t test). (T–Y) Comparison of E12.5 control (*Cxcr4*^*fl/−*^; T and W) with *Tie2-Cre;Cxcr4fl*^*/−*^ (U and X) and *Pax3-Cre;Cxcr4fl*^*/−*^ (V and Y) mutant SLVs, whole-mount immunostained with anti-PECAM-1 antibody (confocal images). endo, endocardial; mes, mesenchymal. Scale bars represent 100 μm. See also Figure S4.

Mispositioning of CXCR4^+^ cells is the earliest manifestation of the *Cxcl12* null phenotype; however, null SLVs also exhibit hyperplasia (Figures 1M and 1N). To delineate the timing of these aspects of the phenotype, we initiated timed inactivation of *Cxcr4* (Figure 5A) using the inducible, ubiquitous *Caggs-Cre*^*ERT*^ line. Induction with tamoxifen at E13.5 resulted in enlarged, thickened SLVs in 5/6 *Caggs-Cre*^*ERT*^*;Cxcr4*^*fl/−*^ E18.5 embryos compared to *Caggs-Cre* ^*ERT*^*;Cxcr4*^*fl/+*^ controls (n = 4; Figures 5D, 5E, S4A, and S4B). Hyperplasia was induced rapidly, as enlarged SLV leaflets were observed 24 h after tamoxifen administration (Figure 5C). However, the overall dysplasia observed was relatively mild compared to constitutive *Cxcl12* nulls and leaflet misalignment was absent. Importantly, the temporal separation of misalignment and severe dysplasia (E11.5–E12.5) and hyperplasia (E13.5 onward) indicates distinct roles of CXCL12 signaling at different stages of SLV development.

To investigate further the requirement for CXCL12/CXCR4 in controlling cell proliferation in SLVs, we assessed EdU incorporation at E12.5 and E15.5 in CXCR4^+^/CXCR4^−^ MCs and ECs (Figures 5F–5J). MC proliferation rates were similar in controls and *Cxcl12* nulls at E12.5. However, although in controls there was a marked decrease in the rate of proliferation of CXCR4^+^ MCs between E12.5 and E15.5 (37.8% in AoV and 41.3% in PV), proliferation of CXCR4^+^ MCs was not significantly reduced in *Cxcl12* nulls at E15.5. In contrast, proliferation of CXCR4^−^ MCs was significantly decreased in both controls (56% in AoV and 69% in PV) and *Cxcl12* nulls (43.2% in the AoV and 51% in the PV). Proliferation rates in endocardial cells were similar in controls and nulls (Figure 5J). Maintenance of an abnormally high proliferation rate in CXCR4^+^ MCs at E15.5 in *Cxcl12* nulls suggests that CXCL12 signaling is required for the downregulation of cell proliferation during SLV maturation and remodeling.

### Roles of CXCR4^+^ cells of different origins in SLV defects

To compare the roles of CXCR4 in SLV cells of differing origins, CXCR4 was conditionally ablated in endocardial and NC lineages using *Tie2-Cre* or *Pax3-Cre*, respectively. Histological analysis of E15.5 conditional mutants showed SLV misalignment, dysplasia, and hyperplasia in 5/6 *Tie2-Cre* conditional mutants (compared to 4 *Tie2-Cre;Cxcr4*^*fl/+*^ controls), whereas 13/13 *Pax3-Cre* conditional mutants exhibited a milder, hyperplastic phenotype characterized by thickening of the hinge regions, without misalignment of the leaflets (compared to 6 *Pax3-Cre;Cxcr4*^*+/−*^ and 2 *Pax3-Cre;Cxcr4*^*fl/+*^ controls; Figures S4C–S4H). Analysis of 3D-reconstructed whole-mount PECAM-1-stained SLVs revealed significant, similar overall increases in total valve volume in *Tie2-Cre* (1.3- and 1.6-fold in AoV and PV, respectively; n = 4) and *Pax3-Cre* (1.5- and 1.3-fold in AoV and PV, respectively; n = 6) conditional nulls (Figures 5K–5S; see also Figure S4I). However, whereas at least one SLV was misaligned in each of 4 whole-mount-immunostained *Tie2-Cre*;*Cxcr4*^*fl/−*^ hearts (p < 0.05), leaflet alignment was normal in all *Pax3-Cre;Cxcr4*^*fl/+*^ SLVs.

At E12.5, misaligned, malformed SLVs were observed in *Tie2-Cre* conditional null hearts only (Figures 5T–5Y). Abnormalities were seen in 4/5 AoVs and 2/5 PVs in *Tie2-Cre*;*Cxcr4*^*fl/−*^ mutants and 0/5 *Pax3-Cre*;*Cxcr4*^*fl/−*^ mutant AoVs or PVs, compared to *Cxcr4*^*fl/−*^ controls (n = 3). These results are consistent with an early role for CXCR4 in the positioning of endocardially derived MCs. Analysis of CXCR4 expression at E13.5 showed a comparable decrease in both types of conditional null (Figures S4J–S4Q’). *Pax3-Cre*;*Cxcr4*^*fl/−*^ SLVs were normal in appearance at E13.5 (n = 3), showing that hyperplasia manifests between E13.5 and E15.5. Thus, whereas CXCR4 expressed in endocardial derivatives is required for the process of leaflet shaping and alignment between E11.5 and E12.5, CXCR4^+^ cells of both endocardial and NC origin contribute to the regulation of leaflet growth at later developmental stages.

### Analysis of *Cxcr7* null phenotype and CXCR7 protein expression

**Figure 6:**
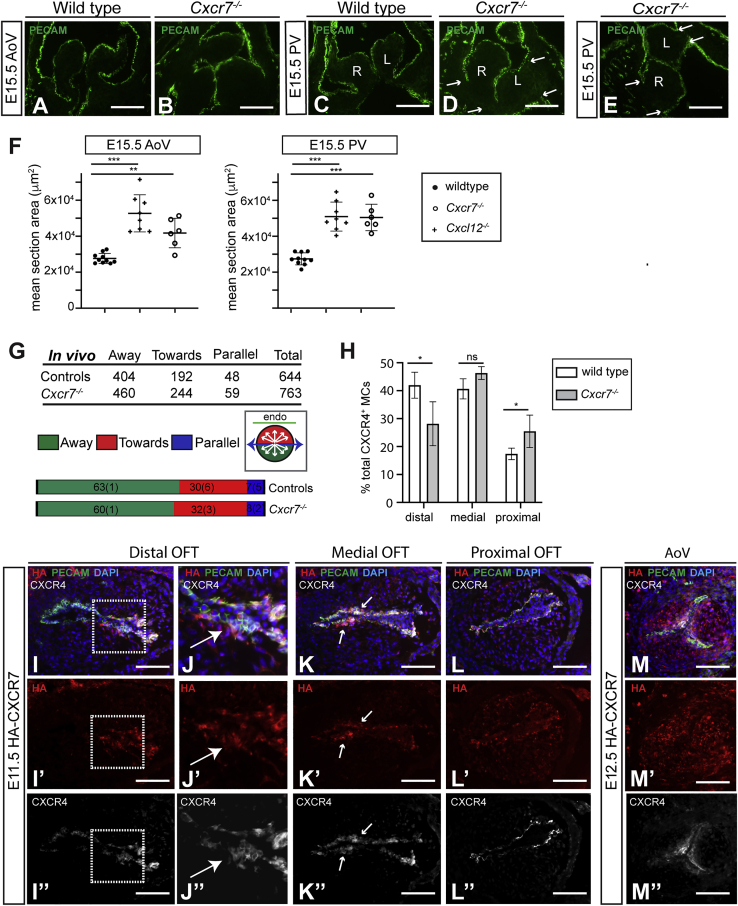
Analysis of *Cxcr7*-null phenotype and CXCR7 protein expression (A–E) Wild-type (A and C) and *Cxcr7*^*−/−*^ SLVs (B, D, and E) immunostained with anti-PECAM-1 antibodies. Arrows in (D) and (E) indicate edges of leaflet bases; misalignment of the left (L) and right (R) leaflets was observed in (E), but not (D). (F) Graphs show mean SLV section area in E15.5 controls, *Cxcl12*^*−/−*^, and *Cxcr7*^*−/−*^ SLVs. Individual values are shown; bars indicate mean ± SD; ^∗∗^p < 0.01 and ^∗∗∗^p < 0.001 (unpaired Student’s t test). (G) Analysis of CXCR4^+^ MC orientation in *Cxcr7*-null and control E11.5 OFT. Graphs compare cell percentages per orientation, standard deviation in brackets; table shows cell numbers. Inset circular diagram indicates criteria for cell orientation. No significant difference between controls and nulls is shown (p = 0.636; chi-squared test). (H) Distribution of CXCR4^+^ MCs in distal, proximal, and medial OFT as a percentage of total CXCR4^+^ MC number, in wild type (n = 4) and *Cxcr7^-/-^* (n = 5). Error bars represent ±SD; ns, non-significant, ∗p ≤ 0.05 (Student’s t test). (I–M’’) Detection of CXCR7 in HA-CXCR7 mice: distal (I–J”), medial (K–K”), and proximal (L–L”) E11.5 OFT and E12.5 AoV sections (M–M”) were co-immunostained with anti-HA (detecting CXCR7), CXCR4 (cl. 2B11; detecting total CXCR4), and anti-PECAM-1 antibodies. Boxed regions in (I)–(I”) are enlarged in neighboring (J)–(J”). Arrows in (K)–(K”) indicate CXCR4^+^ MCs. Scale bars represent 100 μm. See also Figure S5.

CXCR7 can act synergistically, antagonistically, or independently of CXCR4 (reviewed by Sánchez-Martín et al., 2013). As SLV defects have been reported in *Cxcr7* mutants (Sierro et al., 2007; Yu et al., 2011), we examined *Cxcr7*^*−/−*^ hearts to investigate potential interplay between CXCL12, CXCR4, and CXCR7 during SLV development. E15.5 *Cxcr7^-/-^* SLVs were enlarged, with thickened hinges (Figures 6A–6E). Although mean leaflet area was increased to a similar extent in both *Cxcr7* (n = 8) and *Cxcl12* (n = 6) nulls, compared with controls (n = 10; Figure 6F), only 3/16 *Cxcr7^-/-^* SLVs (18.8%; p < 0.05) showed evidence of leaflet misalignment (see Figures 6D and 6E). Co-localization of CXCR4 puncta with the Golgi was preserved in *Cxcr7* nulls at E11.5 (Figures S5A–S5A”). The patterning of SLV primordia appeared broadly normal in *Cxcr7*^*−/−*^ E11.5 OFT (Figures S5B–S5G), and CXCR4^+^ MC orientation was not significantly altered compared to controls (Figure 6G); nevertheless, quantitation of CXCR4^+^ MCs in distal, medial, and proximal sectors of the OFT showed a shift in distribution from distal to proximal (Figure 6H), albeit a smaller and less significant shift than previously observed in *Cxcl12* nulls (see Figure 3K). In addition, *Cxcr7^-/-^* SLVs examined at E12.5 (n = 7) appeared somewhat dysplastic (Figures S5H–S5K). Overall, although the severity of hyperplasia in *Cxcr7*^*−/−*^ SLVs was comparable to *Cxcl12* nulls, other aspects (relating to early effects on cellular orientation and distribution) were milder.

We went on to examine co-localization of CXCR7 and CXCR4 proteins by analyzing tissue sections from mice expressing CXCR7 tagged with hemagglutinin (HA) (Saaber et al., 2019). Both punctate and membranous forms of CXCR7 were detected using an anti-HA antibody (Figures 6I–6M”). At E11.5, HA-CXCR7 was observed in the distal, medial, and proximal OFT, being largely co-expressed with CXCR4 in both ECs and MCs in the valve-forming region (Figures 6I–6L”). However, HA-CXCR7 also appeared in CXCR4^−^ MCs, increasing in a distal-proximal fashion (compare Figures 6I’ and 6L’). At E12.5, HA-CXCR7 was detected in all SLV MCs, thus partially overlapping with CXCR4, and was mainly excluded from endocardium (Figures 6M–6M” and S5L–S5L”). Overall, the level of HA-CXCR7 increased between E11.5 and E12.5. These expression data are compatible with overlapping roles and/or interaction of CXCR7 with CXCR4 during SLV morphogenesis.

### Ablation or inhibition of CXCR7 and excess CXCL12 have similar effects on intracellular localization of CXCR4

**Figure 7:**
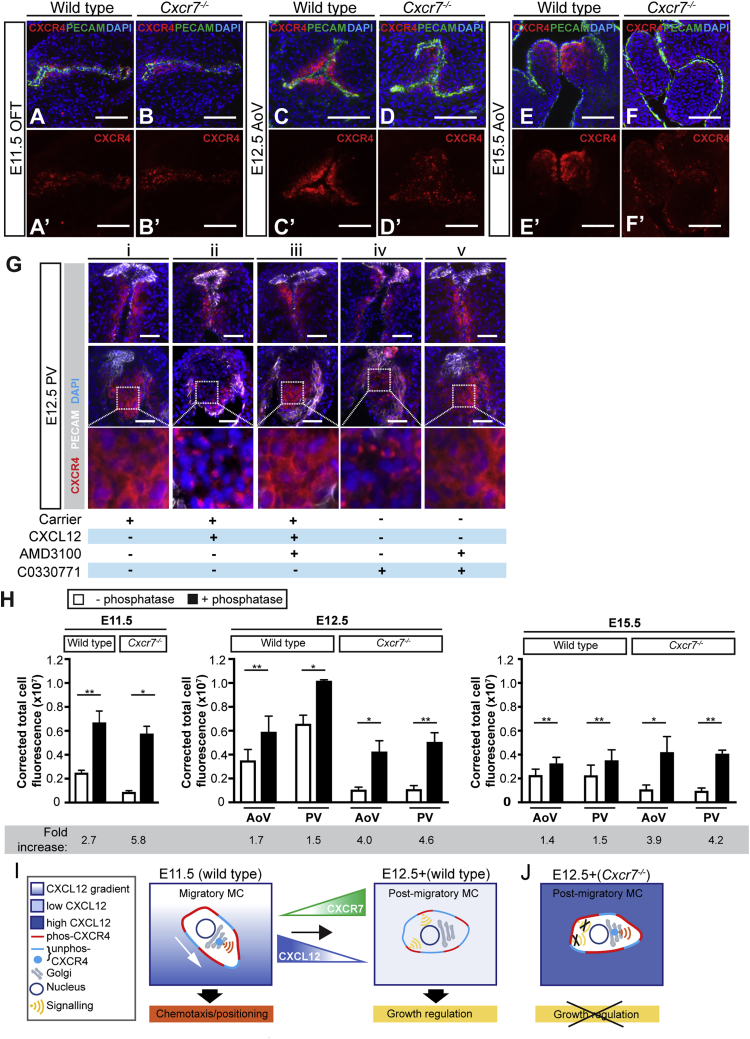
SLV mesenchymal cells are over-exposed to CXCL12 in the absence of CXCR7 (A–F’) E11.5–E15.5 wild-type (A, A’, C, C’, E, and E’) and *Cxcr7*^*−/−*^ (B, B’, D, D’, F, and F’) OFT/AoV sections co-immunostained with anti-CXCR4 and anti-PECAM-1 antibodies. (G) *Ex vivo* treatment of E12.5 hearts (wild type) with carrier only (DMSO; i), CXCL12 (ii), CXCL12/AMD3100 (iii), C0330771 (CXCR7 inhibitor; iv), or CXCL12/C0330771 (v). PV sections (top row, left and right leaflets; middle row, anterior leaflets; bottom row, enlargements of boxed areas) were co-immunostained as above. (H) λPP treatment of wild-type and *Cxcr7*^*−/−*^ OFT/SLV tissue sections: graphs show corrected total cell fluorescence of E11.5–E15.5 serial sections ± λPP immunostained with anti-CXCR4 antibody; fold changes are shown underneath. Error bars represent ± SD; ^∗^p ≤ 0.05 and ^∗∗^p ≤ 0.01 (paired t test). (I and J) Hypothetical model for differential downstream signaling in migratory (E11.5) and post-migratory (E12.5+) CXCR4^+^ MCs (I). Migratory MCs exposed to CXCL12 gradient (graded blue) have high phos-CXCR4 on surface (red) plus punctate (internalized) CXCR4 (unphos, turquoise); signaling downstream of CXCL12/CXCR4 occurs via internal membrane compartment, e.g., Golgi (promoting chemotaxis). Increased CXCR7 between E11.5 and E12.5 reduces both CXCL12 concentration (by sequestration) and CXCR4 internalization; signaling (leading to downregulation of proliferation) occurs via plasma membrane. In *Cxcr7* nulls, increased effective CXCL12 (dark blue) leads to persistent internalization of CXCR4, preventing activation of pathways dependent on signaling from plasma membrane (J). phos, phosphorylated; unphos, unphosphorylated. Scale bars represent 100 μm in (A)–(F’) and 50 μm in (G). See also Figure S6.

Examination of CXCR4 expression in *Cxcr7* nulls revealed that CXCR4 puncta persist after E11.5, with a concomitant reduction in CXCR4 located on the cell surface (Figures 7A–7F’ and S5M–S5P’; n = 4–6 controls/nulls per stage). CXCR4 puncta were not observed in *Cxcl12*^*−/−*^*;Cxcr7*^*−/−*^ SLVs (Figures S5Q and S5Q’), indicating that their presence depended on CXCL12. Formation of CXCR4 puncta is associated with exposure to high levels of CXCL12 (Lapidot and Kollet, 2002; Mimura-Yamamoto et al., 2017). As CXCR7 has a high affinity for CXCL12 and has been shown to sequester CXCL12 in various contexts (Balabanian et al., 2005; Burns et al., 2006; Donà et al., 2013; Naumann et al., 2010), we wondered whether the persistence of CXCR4 puncta could be linked to scavenging of CXCL12 by CXCR7, potentially resulting in abnormally high levels of CXCL12 in *Cxcr7*^*−/−*^ SLVs.

To examine whether CXCL12 uptake occurs in the OFT, we incubated E11.5 and E12.5 wild-type hearts with labeled CXCL12 (biotinylated CXCL12 conjugated to streptavidin-AF594, referred to hereafter as b-CXCL12). At E11.5, speckled b-CXCL12 signal was detected predominantly in ECs (arrows, Figures S6A and S6A’), with some positive underlying MCs medially (asterisk). At E12.5, signal was almost entirely limited to the MCs at the leaflet tips (asterisks in Figures S6C and S6C’). Co-incubation with unlabeled CXCL12 abrogated b-CXCL12 signal (Figures S6B, S6B’, S6D, and S6D’). These findings suggested active internalization of exogenous CXCL12 in the OFT and SLVs.

To examine further the possible connection between CXCR7, CXCR4 puncta, and the level of CXCL12 exposure, we analyzed the intracellular localization of CXCR4 in E12.5 wild-type hearts treated *ex vivo* with CXCL12 and/or inhibitors of CXCR7 (C0330771) or CXCR4 (AMD3100; Figure 7G). Control hearts treated with carrier alone exhibited mainly membranous expression of CXCR4 at E12.5, as expected (n = 4). Treatment with a high dose of CXCL12 (100 μM) resulted in the appearance of CXCR4 puncta and a reduction in membranous CXCR4 (n = 5). This could be prevented by pre-treatment with AMD3100 (n = 3), which reportedly inhibits endocytosis of CXCR4 (Hatse et al., 2002; Hitchinson et al., 2018). Treatment of the hearts with C0330771 (and without addition of CXCL12) was also sufficient to induce CXCR4 puncta (n = 5); again, co-incubation with AMD3100 prevented the appearance of puncta (n = 4). These data therefore support the notion that CXCR4 puncta in the OFT and SLVs result from receptor endocytosis following cellular exposure to high levels of CXCL12. Inhibition of CXCR7 mimics this effect, suggesting that this effectively raises the local concentration of CXCL12.

### Increased phosphorylation of CXCR4 in *Cxcr7* nulls

Phosphorylation of CXCR4 by CXCL12 at multiple C-terminal sites occurs rapidly after activation (Busillo et al., 2010; Busillo and Benovic, 2007), and exposure to elevated CXCL12 has been shown to induce an increase in phosphorylated CXCR4 (phos-CXCR4) on the plasma membrane (Mimura-Yamamoto et al., 2017). We analyzed CXCR4 phosphorylation in *Cxcr7^-/-^* OFT (E11.5) and SLVs (E12.5 and e15.5) in order to assess the level of receptor activation in the absence of CXCR7 (Figures 7H and S6E–S6N’). As CXCR4 antibodies cl. EPUMBR3 and cl. UMB2 detect only unphos-CXCR4, tissue sections were treated with λ protein phosphatase (λPP) before immunostaining, to enable detection of total CXCR4 (unphos-CXCR4 + phos-CXCR4). The difference in immunoreactivity observed between serial sections ± λPP treatment was interpreted as the level of phos-CXCR4. At all stages, λPP treatment increased CXCR4 immunoreactivity in both wild types and *Cxcr7* nulls when compared with untreated sections (Figures S6E–S6N’). In wild types, a 2.7-fold increase was observed after λPP treatment at E11.5, accompanied by detection of a correspondingly higher level of membranous phos-CXCR4; smaller increases (approximately 1.5-fold) occurred at E12.5 and E15.5 following λPP treatment (Figure 7H). This indicated higher levels of phos-CXCR4 (and thus CXCR4 activation) at E11.5 compared with later stages. In *Cxcr7* nulls, larger increases in fluorescence after λPP treatment were observed at E11.5 (5.8-fold), E12.5 (4.0- and 4.6-fold in AoV and PV, respectively) and E15.5 (3.9- and 4.2-fold in AoV and PV, respectively), i.e., more than double the increases seen in wild types (Figure 7H). Thus, a greater proportion of total CXCR4 protein was phosphorylated in *Cxcr7* nulls, and in contrast to wild types, this was maintained post-E11.5. Together with the *ex vivo* data above, the following conclusions can be drawn from these analyses: (1) in wild types, the increased proportion of phos-CXCR4 at E11.5 compared to E12.5 and E15.5, combined with the relative frequency of CXCR4 puncta at E11.5 and E12.5 (see Figure 2I) implies that CXCR4^+^ cells are exposed to a higher level of CXCL12 at E11.5 than at later stages of SLV development. (2) Increased internalization and activation of CXCR4 in the absence of CXCR7 indicate exposure to excess CXCL12, suggesting that sequestration by CXCR7 normally occurs from E12.5 onward. Thus, scavenging of CXCL12 by CXCR7 within SLV primordia may be important for modulating CXCL12-activated pathways during valve development.

## Discussion

This study has identified CXCR4 as a key regulator of cellular migration during endoMT in the valve-forming region of the OFT and revealed distinct roles for CXCL12 signaling at different stages of SLV development, affecting both distribution and proliferation of CXCR4^+^ MCs. Sequestration of CXCL12 by CXCR7 modulates the level of cellular exposure to ligand during these temporally separated processes (Figures 7I, 7J, and S7A).

Our analysis of CXCR4^+^ MCs within both wild-type and *Cxcl12*-null OFT at E11.5 illuminates a critical early phase of SLV morphogenesis following the initiation of endoMT, whereby the patterning of endocardially derived cells forming the SLV anlagen depends on a gradient of CXCL12. Loss of guidance cues in *Cxcl12*-null OFT leads to misorientation and mislocalization of CXCR4^+^ MCs, leading to an overall distal-proximal shift in distribution and defective leaflet shape and alignment. The role of CXCL12 signaling in attractive guidance of multiple cells types is well established (reviewed by Lewellis and Knaut, 2012; Wang and Knaut, 2014); chemokine availability must be tightly regulated in order to facilitate precise navigation of cells, which can occur over relatively long distances and through dynamic environments, e.g., zebrafish posterior lateral line primordium migrate the length of the body (Ghysen and Dambly-Chaudière, 2007) using a self-generated gradient of Cxcl12a for long-range guidance (Donà et al., 2013; Venkiteswaran et al., 2013). However, as the migratory distances for CXCR4^+^ MCs within the OFT are relatively short, the mechanisms that determine their normal distribution within the OFT may be driven at least in part by the shape of the cushions and resulting local differences in CXCL12 concentration (see Figure S7B).

Cells populating the valve-forming region of the OFT cushions at E11.5 are characterized by intracellular CXCR4 puncta that co-localize with the Golgi apparatus, in line with previous studies in human T cells (Kumar et al., 2011) and migrating granule progenitor cells (Mimura-Yamamoto et al., 2017). Receptor internalization is important for downregulating signaling from the plasma membrane but can also serve to compartmentalize or prolong signaling events. Previous studies have shown that CXCR4 internalization is required for the correct migration and positioning of precursor cells (Mimura-Yamamoto et al., 2017; Minina et al., 2007), while the Golgi is known to play an important role in directional cell migration and acts as a signaling hub for several different pathways (Millarte and Farhan, 2012). Furthermore, some GPCRs, e.g., TSH (thryroid stimulating hormone) receptor, have been shown to mediate G protein activation at Golgi-associated membrane compartments (Eichel and von Zastrow, 2018; Godbole et al., 2017; Thomsen et al., 2018). Thus, co-localization of CXCR4 puncta with the Golgi may be functionally significant with regard to CXCL12/CXCR4-dependent signaling pathways. The absence of puncta in E11.5 *Cxcl12* nulls reflects the lack of receptor phosphorylation and internalization in the absence of ligand and suggests a failure to activate appropriate downstream signaling mechanisms. Indeed, our OFT explant invasion assays demonstrate that the ability of CXCR4^+^ MCs to orientate themselves in response to a CXCL12 gradient depends on receptor internalization and subsequent downstream activation of PI3K/AKT (and not MAPK/ERK) signaling (Sorkin and Von Zastrow, 2002, 2009). Furthermore, as CXCR4 puncta are undetectable in the majority of post-migratory CXCR4^+^ valve MCs at E12.5, their presence at E11.5 may reflect a stage-dependent ability to respond to directional cues, dependent on signaling pathways activated from internal membrane compartments rather than from the plasma membrane (Figure 7I). Although further work is required to verify this model, our findings are supported by recent studies suggesting that specific CXCL12-dependent pathways may be activated from different intracellular compartments, e.g., CXCL12-induced phosphorylation of AKT, but not ERK-1/2, was attenuated by inhibition of endocytosis (English et al., 2018). In a separate study, knockdown of clathrin enhanced CXCL12-dependent ERK-1/2 signaling but reduced cell migration *in vitro* (DeNies et al., 2019).

Timed inactivation of CXCR4 showed that the CXCL12/CXCR4 axis has distinct roles at different phases of SLV development. Downregulation of cellular proliferation by CXCL12 signaling appears to be important during the maturation and sculpting phase of SLV development. Our study and others (Aikawa et al., 2006; Goddard et al., 2017; Hinton et al., 2006; MacGrogan et al., 2016) have shown that the rate of MC proliferation declines as SLV development progresses. A failure to downregulate proliferation of CXCR4^+^ MCs between E12.5 and E15.5 leads to hyperplasia in *Cxcl12*-null SLVs, exacerbating an already dysplastic phenotype. Although CXCR4^−^ MC proliferation is significantly reduced in *Cxcl12*^*−/−*^ SLVs within the same time frame, these cells still proliferate more rapidly compared to equivalent cells in controls. This shows that, with respect to CXCR4, non-cell-autonomous mechanisms of growth regulation may also be important. BMP/transforming growth factor β (TGF-β), epidermal growth factor (EGF), and NF1/RAS signaling pathways have all been implicated in driving expansion of endocardial cushion mesenchyme from E11.5 (MacGrogan et al., 2014), while JAG1-NOTCH1 activity (MacGrogan et al., 2016) is required to downregulate MC proliferation later in development. Further work is required to elucidate the pathways that regulate proliferation downstream of CXCL12/CXCR4 in the SLVs.

Although endoMT is an important mechanism underlying development of the SLVs, other sources of cells make significant contributions (Eley et al., 2018; Liu et al., 2018; Nakamura et al., 2006; Phillips et al., 2013). Correspondingly, our lineage-tracing experiments show multiple origins for CXCR4^+^ MCs in the OFT at E11.5, with the majority derived from endocardium and a smaller proportion from SHF and NC lineages. Knockout of *Cxcr4* in the endocardium alone confirmed the critical, early role of endocardially derived CXCR4^+^ cells in shaping the SLVs. The phenotype observed in *Pax3-Cre*-driven CXCR4 conditional nulls, of hyperplasia without gross dysplasia or misalignment, suggests that the role of NC-derived CXCR4^+^ MCs is restricted to post-migratory growth regulation, in keeping with the increased abundance of CXCR4^+^ NC derivatives from E12.5 onward.

CXCR7 expression overlapped with CXCR4 in the OFT at E11.5 but was also more widely expressed. Comparison of the *Cxcr7* and *Cxcl12* null phenotypes indicates a more significant role for CXCR7 in SLV remodeling than in directed cell migration. Nonetheless, the distal-proximal shift in CXCR4^+^ MC distribution observed in *Cxcr7^-/-^* OFTs at E11.5 suggests that CXCR7 may be required to limit the spread of CXCR4^+^ MCs into the proximal OFT. One possibility is that CXCR7 co-activates CXCR4 at this stage (Levoye et al., 2009; Sierro et al., 2007) or it may independently activate downstream signaling required for control of migration (Valentin et al., 2007; Wang et al., 2011). Alternatively, CXCR7 may act to refine the CXCL12 gradient in order to increase the accuracy of cellular migration (Lewellis and Knaut, 2012).

Our *in vivo* and *in vitro* data confirmed an association between CXCR4 puncta and either high levels of CXCL12, or inhibition or absence of CXCR7. The decreased frequency of CXCR4 puncta (and reduction in phosphorylated CXCR4) in wild types coincides with the expansion of SLV mesenchymal CXCR7 expression between E11.5 and E12.5; thus, we surmise that CXCR7 normally sequesters CXCL12 in the SLVs from E12.5 onward. CXCL12 sequestration by CXCR7 is reminiscent of previous studies on several tissues, including mammalian interneurons and zebrafish primordial germ cells and lateral line primordium (Abe et al., 2014; Boldajipour et al., 2008; Dambly-Chaudière et al., 2007; Memi et al., 2013; Naumann et al., 2010; Saaber et al., 2019; Venkiteswaran et al., 2013). However, we hypothesize that, rather than generating or refining a CXCL12 gradient to guide migratory cells as observed in these tissues, sequestration of ligand by CXCR7 in post-migratory valve MCs may instead function to limit CXCR4 internalization after the earliest, endoMT-associated phase of SLV development. This may reduce or abrogate activation of downstream pathways regulating cellular migration required during endoMT (potentially activated from internal membrane compartments, e.g., Golgi) and instead favor activation of pathways regulating cellular proliferation (potentially activated from the plasma membrane) required during the leaflet remodeling phase (Figure 7I). Conversely, CXCR7 ablation leading to continued high levels of CXCL12 may maintain activation of the earlier pathways at the expense of pathways normally activated from E12.5 onward, leading to dysregulated proliferation (Figure 7J). This putative signaling switch and its mechanism, which differ from previously described models of CXCR7 activity, warrant further investigation. However, we cannot exclude the additional possibility that CXCR7 may regulate SLV growth directly by activating downstream signaling independent of CXCR4 (Rajagopal et al., 2010; Wang et al., 2011) or indeed G proteins (Heuninck et al., 2019). Alternatively, CXCR7 may heterodimerize with CXCR4 to activate divergent pathways (Décaillot et al., 2011; Heuninck et al., 2019; Levoye et al., 2009; Sierro et al., 2007). As modulation of CXCL12 signaling can also be achieved by differential phosphorylation of the C-terminal serines of CXCR4 (Busillo et al., 2010), the overall picture of CXCL12/CXCR4/CXCR7 signaling is complex and the role of CXCR7 is likely to be highly context dependent.

In conclusion, we have demonstrated dual roles for CXCL12 signaling in SLV morphogenesis and shown that CXCR7 can modulate CXCL12 availability at different developmental stages. In theory, this provides a mechanism for the activation of divergent downstream pathways by a single chemokine in one tissue. We have previously shown that CXCL12 signaling directs the formation of the peritruncal plexus from E11.5 (Ivins et al., 2015) by guiding CXCR4^+^ endothelial cells toward the lumen of the OFT, distal to the endocardial cushions. CXCR7 may be key to the precise orchestration of two opposed migratory processes occurring in close proximity within a single structure, through its capacity to tightly control the spatial and temporal distribution of CXCL12. The role of CXCL12 signaling in the OFT serves to illustrate the versatility of the CXCL12 signaling axis, whose modes of action appear to be exquisitely tailored to suit the needs of particular tissues and organisms.

### Limitations of the study

Future studies could be usefully directed at exploring further how CXCL12 regulates proliferation of CXCR4^+^ mesenchymal cells and how CXCR7 affects the CXCL12-mediated proliferation. The pathway analysis that is included here is limited to *ex vivo* explant models where we may lose potentially important interactions. In addition, we have not explored how the CXCL12 pathway cross-talks with important flow-mediated sculpting events vital for achieving normal valve morphogenesis.

## STAR★Methods

### Key resources table

**Table:** 

REAGENT or RESOURCE	SOURCE	IDENTIFIER
**Antibodies**		

Armenian hamster monoclonal anti-PECAM-1 (Clone 2H8)	ThermoFisher	Cat#MA3105; RRID:AB_223592
Rabbit monoclonal anti-CXCR4 (Clone EPUMBR3, unphosphorylated)	Abcam	Cat#ab181020; RRID: N/A
Rabbit monoclonal anti-CXCR4 (Clone UMB2, unphosphorylated, identical epitope to Clone EPUMBR3)	Abcam	Cat#ab124824; RRID:AB_10975635
Rat monoclonal anti-CXCR4 (Clone 247506)	R&D Systems	Cat#MAB21651; RRID:AB_2261636
Rat monoclonal anti-CXCR4 (Clone 2B11)	Invitrogen	Cat#14-9991-82; RRID:AB_842770
Chicken polyclonal anti-GFP	Abcam	Cat#ab13970; RRID:AB_300798
Rabbit monoclonal anti-GM130 (Clone EP892Y)	Abcam	Cat#ab52649; RRID:AB_880266
Mouse monoclonal anti-GM130 (Clone 35/GM130)	BD Transduction Laboratories	Cat#610822; RRID:AB_398141
Mouse monoclonal anti-g-tubulin (Clone GTU-88)	Sigma	Cat#T6557; RRID:AB_477584
Rabbit monoclonal anti-HA-Tag (Clone C29F4)	Cell Signaling Technology	Cat#3724; RRID:AB_1549585
Rabbit monoclonal anti-SLUG (Clone C19G7)	Cell Signaling Technology	Cat#9585; RRID:AB_2239535
Mouse monoclonal anti-SOX9 (Clone GMPR9)	Invitrogen	Cat#14-9765-80; RRID:AB_2573005
Goat anti-rabbit, AF594 conjugate	Invitrogen	Cat#A11037; RRID:AB_2534095
Goat anti-rabbit, AF647 conjugate	Invitrogen	Cat#A21245; RRID:AB_2535813
Goat anti-armenian hamster, AF488 conjugate	Abcam	Cat#ab173003; RRID: N/A
Goat anti-armenian hamster, AF647 conjugate	Abcam	Cat#ab173004; RRID:AB_2732023
Donkey anti-rat, AF488 conjugate	Invitrogen	Cat#A21208; RRID:AB_2535794
Goat anti-chicken, AF488 conjugate	Invitrogen	Cat#A11039; RRID:AB_2534096
Goat anti-mouse IgG1, AF594 conjugate	Invitrogen	Cat#A21125; RRID:AB_2535767

**Chemicals, peptides, and recombinant proteins**		

Murine recombinant CXCL12	R&D Systems	Cat#350-NS
AMD3100 (reconstituted at 2mM in sterile water, working concentration of 10μM)	Generon	Cat#A13074
LY294002 (reconstituted at 10mM in DMSO, working concentration of 10μM)	Cell Signaling	Cat#9901
U0126 (reconstituted at 10mM in DMSO, working concentration of 10μM)	Cambridge Bioscience	Cat#SM106-5
Dynasore (reconstituted at 80mM in DMSO, working concentration 80μM)	Abcam	Cat#ab120192
C0330771 (reconstituted at 2mM in DMSO, working concentration 10μM)	Dr Thomas Schall, Chemocentryx	N/A
Biotinylated CXCL12	Generon	Cat#OPCB00014
Streptavidin	BioLegend	Cat#405150
Streptavidin, AF594 conjugate	Invitrogen	Cat#S11227
Collagen type 1, high concentration, rat tail	VWR	Cat#734-1085
Lambda protein phosphatase	New England Biolabs	Cat#P0753S

**Critical commercial assays**		

Click-iT EdU Cell Proliferation Kit	Invitrogen	Cat#C10339
Alcian Blue Stain Kit	Vector	Cat#H-3501
Mouse on Mouse (M.O.M®) Basic Kit	Vector Laboratories	Cat#BMK-2202

**Experimental models: organisms/strains**		

Mouse: Cxcl12-GFP: B6.129P2-Cxcl12 < tm2Tng > /TngRbrc	RBRC	Stcok#RBRC04200
Mouse: HA-Cxcr7	Ralf Stumm, Jena University Hospital; Germany	Saaber et al., 2019
Mouse: Tie2-Cre: B6.Cg-Tg(Tek-cre)1Ywa/J	The Jackson Laboratory	Stock#008863
Mouse: Mef2c-Cre: Tg(Mef2c-cre)2Blk/Mmnc	MMRRC	Stock#030262-UNC
Mouse: Pax3-Cre: B6.129-Pax3 < tm1(cre)Joe > /J	The Jackson Laboratory	Stock#005549
Mouse: Cxcr4-flox: B6.129P2-Cxcr4 < tm2Yzo > /J	The Jackson Laboratory	Stock#008767
Mouse: Cxcr4 ^−/−^	Ivins et al., 2015	N/A
Mouse: ROSA-YFP: B6.129X1-Gt(ROSA)26Sor < tm1(EYFP)Cos > /J	The Jackson Laboratory	Stock#006148
Mouse: Cxcr7-flox: B6;129S-Ackr3 < tm1Twb > /Mmucd	MMRRC	Stock#036715-UCD
Mouse: β-actin-Cre: FVB/N-Tmem163 < Tg(ACTB-cre)2Mrt > /J	The Jackson Laboratory	Stock#003376
Mouse: CAGGS-Cre: B6.Cg-Tg(CAG-cre/Esr1^∗^)5Amc/J	The Jackson Laboratory	Stock#004682

**Oligonucleotides**		

See Table S1		N/A

**Software and algorithms**		

FIJI		https://imagej.net/software/fiji
GraphPad Prism 8	GraphPad Software	https://www.graphpad.com/scientific-software/prism/

**Other**		

Dulbecco’s modified Eagle media (DMEM)	ThermoFisher	Cat#61965-026
Boyden chamber inserts	Sarstedt	Cat#83.3932.040

### Resource availability

#### Lead contact

Requests for further information should be directed to the lead contact, Sarah Ivins (s.ivins@ucl.ac.uk).

#### Materials availability

This study did not generate new unique reagents. There are restrictions to the availability of the *Cxcr7*^flox/flox^ and *Cxcr7*^+/−^ lines (latter derived from the former) due to the presence of an MTA from https://www.mmrrc.org/, and the *Cxcl12*^GFP/+^ line to an MTA from Frontier Medical Sciences Kyoto University (contact Prof Takashi Nagasawa).

### Experimental model and subject details

#### Mice

All animal work was undertaken in accordance with UK Home Office regulations. Individual mouse lines are listed in the Key resources table. All mouse lines were maintained on a C57BL/6 background. Animals were housed in temperature-controlled conditions (20-23°C), in a 12-hour light/dark cycle, with free access to food and water. Timed matings were performed and pregnancy determined by the presence of a vaginal plug. Noon on the day after mating was considered E0.5. Embryonic (E10.5-18.5) sample stages are indicated in the main text and appropriate figure legends. For experiments utilizing the inducible CAGGS-Cre line, recombination was induced by administration of 100μL 20mg/mL tamoxifen (Sigma, T5648) dissolved in glyceryl trioctanoate (Sigma, T9126) to pregnant females by intraperitoneal injection. For all experimental analysis, all animals/embryos were utilized, irrespective of sex, and grouped according to genotype. Transmission of all Cre alleles was achieved through the paternal line.

#### Primary Cell Culture

Pregnant females were sacrificed by cervical dislocation and embryos were harvested at E10.5. Dissected outflow tract explants were grown at 37°C, 5% CO_2_ in Dulbecco’s Modified Eagle Medium (DMEM) (ThermoFisher, 61965-026) supplemented with 10% fetal bovine serum (FBS) (GIBCO, 10270106) and penicillin/streptomycin (GIBCO, 15140-122) unless otherwise stated. OFTs from both male and female embryos were utilized and grouped for downstream treatment based on genotype alone.

### Method details

All protocols were performed at room temperature, unless otherwise stated.

#### Genotyping

DNA samples for genotyping were extracted and purified from embryonic yolk sacs (E ≤ 11.5) or tail clippings (E ≥ 12.5) using standard methods. Briefly, tissue was digested overnight at 56°C in 400 μL lysis buffer (100mM NaCl, Sigma, S7653), 10mM TRIS (Sigma, ST6066), 10mM EDTA (Fisher, BP120), 0.5% SDS (Sigma, 62862), 400ug/mL proteinase K (Sigma, P6556)), and DNA was precipitated by addition of an equal volume of isopropanol (Fisher, P/7490/17). After centrifugation (30 minutes at 17,000G), DNA pellets were washed with 70% (v/v) ethanol (Sigma, 32221), and then resuspended in an appropriate volume of ddH_2_O (typically 100-150 μL). PCRs were set up with BIOTAQ (Bioline, BIO-21040), Amplitaq Gold 360 (Applied Biosystems, 4398876) or KAPA2G Taq (KAPA, KK5700) polymerase kits, as per the manufacturer’s instructions. Genotyping primer pairs are detailed in Table S1. PCR products were subjected to gel electrophoresis on 2% (w/v) agarose (Invitrogen, 16500) gels to determine amplicon sizes.

#### Fixation of Embryonic Hearts

Whole-mount hearts were fixed in 4% paraformaldehyde (PFA) (Sigma, 158127) for 15-30 minutes. For tissue sections, hearts were fixed in 4% PFA for 15-30 minutes (for cryosections) or overnight (for paraffin sections). All specimens were subsequently thoroughly washed with PBS to remove excess PFA. To prepare samples for paraffin embedding, samples were dehydrated through an increasing ethanol concentration series (50/70/85/95/100% (v/v) ethanol in PBS), washed twice with 100% Histo-Clear (National Diagnostics, HS-200), and then moved to 50/50 Histo-Clear/paraffin at 65°C. Samples were then washed with at least three changes of 100% paraffin (RA Lamb, 12624077). All incubations were for 30-60 minutes. For cryo-embedding, samples were first incubated overnight at 4°C in 30% (w/v) sucrose (Fisher, S/8600/53), The following day, samples were incubated in 50/50 30% sucrose/optical cutting temperature compound (OCT) (CellPath, KMA-0100-00A) for 30 minutes, followed by 3 washes in 100% OCT; samples were then snap-frozen in 100% OCT.

#### Histology

Paraffin sections (10μ) were dewaxed in Histo-Clear (2 × 10 minutes), then rehydrated through a decreasing ethanol gradient (2 × 100%, 1 × 90/70/50/30% in ddH_2_O, 2 minutes each) and washed in dH_2_O for 2 × 5 minutes. For H&E staining, slides were incubated in hematoxylin (Sigma, MHSS32) for 3 minutes and then rinsed in at least 3 changes of tap water for a total of ten minutes. Next, slides were incubated in eosin (Sigma, HT110232) for 90 s, washed in ddH_2_O for 2 minutes, and allowed to dry overnight. The next day, coverslips were mounted using DPX mounting media (Millipore, 100579).

Alcian blue staining was performed using an alcian blue staining kit (Vector, H-3501). Briefly, sections were incubated in alcian blue solution (pH2.5) for 30 minutes and counter stained with nuclear fast red, as per the manufacturer’s instructions. Sections were imaged using a Zeiss Axioplan2 Brightfield microscope with Zeiss Axiocam HRc color camera.

#### Wholemount Immunostaining and Volume Measurements

E15.5 hearts were permeabilised in PBS with 0.1% (v/v) Tween 20 (Sigma, P1379) (PBST), blocked for one hour in PBST with 10% goat serum (Sigma, G9023), and incubated in anti-PECAM-1 primary antibody (see Key resources table) diluted 1:400 in blocking buffer overnight at 4°C. Hearts were washed multiple times in PBST (1 hour each) and then incubated in anti-hamster secondary antibody (1:500) overnight at 4°C. After multiple washes with PBST (1 hour each), samples were dehydrated through an increasing methanol (Fisher, M/4058/21) concentration series (25/50/75/3 × 100% in PBS) for 5 minutes each. Samples were then cleared by incubating in 50/50 methanol/BABB (50% Benzyl alcohol (Sigma, 402834)/50% Benzyl Benzoate (Sigma, B6630) for 10 minutes, and then 100% BABB for 10-20 minutes. After replacing the BABB solution, cleared hearts were imaged on a Zeiss LSM 880 upright confocal multiphoton microscope using the multiphoton laser and the 10x/NA0.5 W-Plan Apochromat Water dipping objective. Images were exported to Imaris Image Analysis software (Bitplane) for the manual segmentation of valve leaflets, assembly of 3D reconstructions and measurement of leaflet volumes.

#### General Immunohistochemistry and Quantification

Immunolabelling was carried out using standard protocols. Briefly, frozen tissue sections (10μ) were permeabilised in 0.5% (v/v) Triton X-100 (Sigma, T6878) for 5 minutes before blocking (10% goat serum, 1% BSA (Sigma, A4503), 0.1% (v/v) Triton X-100) for 1 hour. Tissue sections were incubated in primary antibody diluted in blocking buffer overnight at 4°C. Antibodies (see Key resources table) were used at the following dilutions: anti-PECAM-1 (1:400), anti-unphosphorylated CXCR4 (Clone UMB2, 1:300, Clone EPUMBR3, 1:500), anti-CXCR4 (Clone 2B11, 1:200), anti-CXCR4 (Clone 247506, 1:100); anti-GM130 (Clone 35/GM130, 1:300), anti-GFP (1:500) anti-SLUG (1:200), anti-COLLAGEN 1 (1:500), anti-PERIOSTIN (1:300), anti-TWIST (1:50), anti-SOX9 (1:200), anti-HA-Tag (1:500), anti-SM22α (1:250), and anti-αSMA (1:200). The next day, sections were incubated in the relevant secondary antibody (see Key resources table) diluted 1:500 in blocking buffer for 1 hour. Sections were then counter stained with DAPI and coverslips were mounted using CityFluor AF1 mounting media (CitiFluor, 17970). Images were acquired on a Zeiss Axioimager Z1 microscope, exported to FIJI (https://imagej.net/software/fiji ) and processed for analysis and publication. N.B. CXCR4 antibody clones UMB2 and EPUMBR3 were used interchangeably to detect unphosphorylated CXCR4; clones 247506 and 2B11 detect total CXCR4. Unless otherwise stated in the Figure Legends, anti-CXCR4 antibody refers to clones UMB2 or EPUMBR3.

#### Assessment of Leaflet Alignment

SLV leaflet alignment was determined by examining consecutive coronal sections or z stack images (when analyzing confocal images). As alignment of the non-coronal or anterior leaflets in relation to the other leaflets was more likely to be obscured by skewing of the section or imaging plane, alignment of the left and right leaflets only was assessed in order to avoid misleading results. Leaflets were counted as misaligned when the bottom and top edges of the base of one leaflet were displaced by more than half the width of the base of the opposing leaflet in at least 3 consecutive sections.

#### Analysis of Cell Polarity in Tissue Sections

Frozen tissue sections were immunostained with anti-GM130 (mouse monoclonal), anti-CXCR4 (clone UMB2) and anti-PECAM-1 antibodies, as described in ‘General Immunohistochemistry and Quantification’, with the modification that Mouse on Mouse (M.O.M®) Basic Kit (Vector, BMK-2202) reagents were used for blocking sections and dilution of antibodies. Stained sections were imaged on an inverted Zeiss LSM 710 confocal using the 20x Plan Apochromat dry objective. Confocal stacks of sections were exported to FIJI and analyzed to assign orientation to individual CXCR4-positive cells. Positioning of the Golgi and nucleus was used to identify the anterior of cells and their orientation with respect to the nearest endocardial surface. Cell orientation was assigned depending on the angle between a straight line bisecting the Golgi/nucleus of each cell, and the plane of the endocardium, according to the 180 degree sectors illustrated in Figure 3S. The number of cells in each direction category was calculated as a proportion of the total number of CXCR4 positive cells per section analyzed.

For *Cxcl12* nulls and controls, 3-6 sections from 4 embryos of each genotype were analyzed;

for *Cxcr7* nulls and controls, 4-6 sections from 3 embryos per genotype were analyze.

#### OFT Explant Culture

OFT explant cultures were prepared using standard protocols with some modifications (Di Martino et al., 2017; Plein et al., 2015). Briefly, outflow tracts from E10.5 wild-type embryos were dissected into DMEM (+10% FBS), teased open to expose the endocardium, and seeded endocardial side down onto the surface of a 2mg/mL collagen 1 gel (VWR, 734-1085) cast within modified Boyden chamber inserts (Sarstedt, 83.3932.040). Explants were left to adhere to the collagen in the absence of media for 4-5 hours at 37°C. Inserts were then placed in a 24-well plate containing 800 μL DMEM (+10% FBS) per well and cultured overnight at 37°C. After 24 hours, the media in the well was replaced, and 100 μL DMEM (+1% FBS) was added to the insert itself. Explants were cultured for a further 48 hours to allow cells to invade the 3D collagen matrix. Immediately prior to the creation of a CXCL12 gradient, OFT explants were removed, and the remaining cells embedded within and on top of the gel were washed twice with serum free DMEM to remove cell debris and neutralise any remaining chemokine gradient. In order to establish a chemoattractant gradient within the Boyden chamber set-up, 100 μL serum free DMEM was added to the insert, and either 800 μL DMEM supplemented with either PBS + 0.1%BSA (control) or 100nM CXCL12 (R&D Systems, 350-NS) (experimental gradient) was added to the appropriate well. For inhibitor experiments, cells were incubated with DMEM containing either inhibitor (AMD3100, LY294002, U0126, Dynasore) or vehicle (DMSO, water) at the concentration indicated in the Key resources table, for 5 minutes at 37°C, immediately prior to the addition of CXCL12 to the appropriate wells. Note: It is essential that the media within the well is in contact with the insert membrane to permit the passive diffusion of chemokine into the collagen gel. After incubation for 1 hour, gels were washed twice with PBS, fixed in 4% PFA for 10 minutes, and then carefully removed from the insert. Excised gels were washed with PBS, incubated in OCT overnight at 4°C, and then embedded in fresh OCT for cryosectioning, as previously described in ‘Fixation of Embryonic Hearts’.

#### OFT Explant Immunocytochemistry and Cell Polarity Analysis

For immunostaining, slides were treated as described in the ‘General Immunohistochemistry and Quantification’ with some modifications. Briefly, all solutions were carefully aspirated from slides to reduced mechanical disruption and prevent displacement of sections. In addition, post-antibody incubation washes were reduced to one change of PBS for a total period of 10 minutes. Gel sections were blocked using Dako peptide free blocking solution (Dako, X0909), and stained with anti-GM130 (Clone EP892Y, 1:100) and anti-CXCR4 (Clone 247506, 1:100). Images were acquired on a Zeiss Axiovert confocal microscope with Perkin Elmer Ultraview CSU22 spinning disk using a 40x Plan Apochromat oil immersion objective. Images were exported to FIJI and processed for analysis and publication. Cell polarity was assessed as described in ‘Analysis of Cell Polarity in Tissue Sections’; cell orientation was assigned depending on the angle between a straight line bisecting the Golgi/nucleus of each cell, and the plane of the gel surface, according to the sectors illustrated in Figure 4B. The number of cells directed away from the gel surface was calculated as a proportion of the total number of cells per explant analyzed. Cells where the Golgi was not detected were excluded.

#### EdU Incorporation and Quantification

EdU (5-ethynyl-2'-deoxyuridine) incorporation experiments were conducted using the Click-iT EdU Cell Proliferation kit (Invitrogen, C10339) and detected as per the manufacturer’s instructions. Briefly, pregnant females were injected with 200 μL 10uM EdU (in PBS) at the relevant developmental stage. Embryos were harvested 75 mins later and processed for immunostaining with anti-PECAM-1 and anti-unphosphorylated CXCR4 antibodies, as previously described in ‘General Immunohistochemistry and Quantification’. Positive cells displaying EdU incorporation (AlexaFluor 594 signal) were counted in FIJI and calculated as a proportion of i) total CXCR4^+^ mesenchymal (PECAM-1^-^) cells, ii) total CXCR4^-^ mesenchymal cells (PECAM-1^-^) or iii) total endocardial (PECAM-1^+^) cells within the semilunar valve leaflets. 4 *Cxcl12* nulls were compared to 4 controls (wild-type/heterozygous) at E12.5, and 4 *Cxcl12* nulls were compared to 5 controls (wild-type/heterozygous) at E15.5; 4-6 sections were analyzed per embryo.

#### Biotinylated CXC12 Uptake

Hearts from wild-type E11.5 or E12.5 embryos were dissected into PBS (GIBCO, 14190-094). Following a brief incubation in PBS + 2% FBS at 37°C, tissue was blocked in DMEM containing 3.78uM unlabelled streptavidin (BioLegend, 405150) for 30 minutes at 37°C. Samples were washed twice in PBS + 2% FBS, and then incubated in media containing 200nM biotinylated-CXCL12 (Generon, OPCB00014) conjugated to streptavidin-594 (Invitrogen, S11227) for 30 minutes at 37°C. The biotinylated-CXCL12: streptavidin-594 conjugate was formed by incubating these components in a 1:4 molar ratio at room temperature for 3 minutes immediately prior to addition to the tissue, as per manufacturer’s instructions. For competition experiments, samples were co-incubated with 2μM unlabelled CXCL12 (R&D Systems, 350-NS). Samples were washed twice in ice cold DMEM, blocked with PBS + 0.5% BSA for 5 minutes at 4°C, fixed with 4% PFA for 10 minutes, and then washed twice with PBS at 4°C. Specimens were then embedded in OCT for cryosectioning and immunohistochemistry with anti-PECAM-1 antibody as described in ‘General Immunohistochemistry and Quantification’ with the exception that blocking was performed using PBS + 2%BSA. Images were acquired on a spinning disk microscope, as detailed above. Images were exported to FIJI and processed for publication.

#### Ex vivo Treatment of E12.5 Hearts

Hearts were dissected from E12.5 wild-type embryos into pre-warmed PBS, then briefly incubated in 1ml PBS + 2% FBS at 37°C. The hearts were then placed into Eppendorf tubes containing 200 μl DMEM supplemented with inhibitor (AMD3100 or C0330771 at 10μM, see Key resources table) or carrier (DMSO/water) and incubated at 37°C with periodic agitation of the Eppendorf tubes. After 15 minutes the medium was aspirated and replaced with DMEM containing 200nM biotinylated-CXCL12 (see above) or water and the inhibitor/carrier used in the first incubation. The tubes were then incubated for a further hour at 37°C with periodic agitation of the tubes (every 5 minutes). Samples were washed (twice in ice cold DMEM), blocked with PBS + 0.5% BSA for 5 minutes at 4°C, and then fixed with 4% PFA for 10 minutes. After washing twice in PBS at 4°C, samples were then incubated in 30% sucrose overnight at 4°C, prior to embedding in OCT. Immunostaining of cryosections with anti-PECAM-1 and CXCR4 (clone EPUMBR3) antibodies was carried out as described in ‘General Immunohistochemistry and Quantification’.

#### Lambda Protein Phosphatase Treatment and Fluorescence Quantification

Serial frozen tissue sections were incubated at room temperature for 3 hours in the presence or absence of lambda protein phosphatase (800u/slide) (NEB, P0753S) diluted in the supplied buffer (supplemented with MnCl_2_) according to the manufacturer’s instructions. Sections were then washed, blocked and immunostained using anti-PECAM-1 and anti-unphosphorylated CXCR4 antibodies (clone EPUMBR3), and imaged as described in ‘General Immunohistochemistry and Quantification’. To quantify fluorescent intensity, the region of interest (including all CXCR4 signal) was outlined and measured in FIJI; corrected total cell fluorescence (CTCF) intensity of cells was calculated by using the measure tool in FIJI and the following equation (Martin Fitzpatrick, University of Birmingham, UK, available at http://theolb.readthedocs.io/en/latest):

CTCF= integrated density of selected cell – (area of selected cell x mean fluorescence of background)

Four background measurements were used to calculate the background mean fluorescence value for each tissue section analyzed. Cell intensities were compiled from 5-6 pairs of serial sections per embryo; at least 3 embryos were analyzed per stage.

### Quantification and statistical analysis

#### Quantification

##### CXCR4^+^ Cell Distribution

Numbers of CXCR4^+^ MCs were counted in distal, medial and proximal OFT sections (transverse) from stage-matched E11.5 wild-type and mutant embryos (*Cxcl12*^*−/−*^ and *Cxcr7*^*−/−*^*)* and presented as a percentage of total CXCR4^+^ MCs. Sections were stained with anti-PECAM-1 and anti-CXCR4 (clone EPUMBR3) antibodies and DAPI, and CXCR4^+^/PECAM-1^-^ cells were counted using the Fiji Cell Counter plugin. OFT sectors were determined by dividing the OFT (usually 26-34 10μm sections in total, starting from the distalmost section with visible endocardial cushion) into roughly equal thirds, and using OFT morphology to fine-tune the sector boundaries. Equal numbers of sections (4-5) were analyzed per sector, ensuring also that the sections used for analysis were spread evenly along the length of the individual sector. 4 stage-matched controls were compared to 5 nulls for both *Cxcl12*^*−/−*^ and *Cxcr7*^*−/−*^ OFTs.

Numbers of CXCR4^+^ MCs and ECs and their distribution within the OFT (wild-type, Figure 2E) were analyzed similarly (n = 4, 12-15 sections per embryos, > 2000 cells quantitated per embryo).

##### CXCR4 puncta counts

Sections from wild-type E11.5 or E12.5 hearts were stained with anti-PECAM-1 and anti-CXCR4 (clone EPUMBR3) antibodies and DAPI. For E11.5 hearts (n = 4), OFT sectors (distal, medial and proximal) were determined as above. Numbers of CXCR4^+^/PECAM-1^-^ cells with CXCR4 puncta were counted using the Fiji Cell Counter plugin as above and presented as a percentage of the total number of CXCR4^+^/PECAM-1^-^ cells (i.e., including cells with membranous CXCR4 only) per sector. Equal numbers of sections (4-5) were analyzed per sector, as above. For this comparison, distal and medial sector cell counts were merged to give a ‘valve-forming region’ count. For E12.5 hearts (n = 4), 5-6 sections per SLV were analyzed.

##### Leaflet Area measurement

Cryosections of E15.5 hearts were stained with anti-PECAM-1 antibody and DAPI. Where possible, for comparison purposes, sections from null (*Cxcl12*^*−/−*^ or *Cxcr7*^*−/−*^*)* and pooled littermate controls (wild-type or heterozygous mutant) were matched for dorso-ventral position within the SLV. 9-12 coronal sections per SLV were outlined and measured in Fiji; total leaflet area was measured for each section and mean values calculated for controls and nulls. A total of 10 controls, 8 *Cxcr7*^*−/−*^ and 6 *Cxcl12*^*−/−*^ were analyzed.

All other procedures used for quantification are provided in the relevant section of Method details.

#### Statistical analysis

Statistical tests (Student’s t test, Mann Whitney test, Chi squared, one-way ANOVA test) were calculated using GraphPad Prism versions 7-8; tests used are indicated in Figure Legends. Contingency tables were used when comparing numbers of individual defects e.g., alignment defects in mutants and controls, and P values calculated using Fisher’s exact test (https://www.graphpad.com). All data presented in histograms are presented as mean and standard deviation. Significance levels are defined as p < 0.05 (^∗^), p < 0.01 (^∗∗^), p < 0.001 (^∗∗∗^) and p < 0.0001 (^∗∗∗∗^). Details of the statistical test employed, significance levels, and n numbers are provided in the relevant figure legends.

## Data Availability

• Data are available from the lead contact on request. • This study did not generate custom code. • Any additional information required to reanalyse the data reported in this paper is available from the lead contact upon request.

## Supplemental information

Document S1. Table S1 and Figures S1–S7

Document S2. Article plus supplemental information
