# Supplementary material for: Dual role for CXCL12 signaling in semilunar valve development
Source: Cell Rep. 2021 Aug 24;36(8):109610. doi: 10.1016/j.celrep.2021.109610 (PMC8411116; doi:10.1016/j.celrep.2021.109610)
Supplement: Document S1. Table S1 and Figures S1–S7 [file mmc1.pdf]

Cell Reports, Volume *36*

## Supplemental information

# Dual role for CXCL12 signaling

# in semilunar valve development

**Liam A. Ridge, Dania Kewbank, Dagmar Schütz, Ralf Stumm, Peter J. Scambler, and Sarah Ivins**

## Table S1, related to Star Methods

| REAGENT or RESOURCE | SOURCE | IDENTIFIER |
|---|---|---|
| Oligonucleotides (5’>3’) | | |
| Cre Forward: GAACCTGATGGACATGTTCAGG | Qiagen | https://www.qiagen.com/media/ebooks/epaper-Application_Guide_QIAxc/page36.html#/34 |
| Cre Reverse: AGTGCGTTCGAACGCTAGAGCCTGT | As above | As above |
| Myogenin Forward: TTACGTCCATCGTGGACAGC | As above | As above |
| Myogenin Reverse: TGGGCTGGGTGTTAGCCTTA | As above | As above |
| ROSA-YFP Wild Type Forward: GGAGCGGGAGAAATGGATATG | Ivins et al., 2015 | N/A |
| ROSA-YFP Tg Forward: GCGAAGAGTTTGTCCTCAACC | Ivins et al., 2015 | N/A |
| ROSA-YFP Common Reverse: AAGTCGCTCTGAGTTGTTAT | Ivins et al., 2015 | N/A |
| Cxcr4 Recombined Forward: CACTACGCATGACTCGAAATG | Ivins et al., 2015 | N/A |
| Cxcr4 Recombined Reverse: CGGAATGAAGAGATTATGCAGC | Ivins et al., 2015 | N/A |
| Cxcr4 Flox Forward: CCACCCAGGACAGTGTGACTCTAA | The Jackson Laboratory | Primer#10378 |
| Cxcr4 Flox Reverse: GATGGGATTTCTGTATGAGGATTAGC | The Jackson Laboratory | Primer#10379 |
| Cxcr7 Flox Forward: TCTATCGCCTTCTTGACGAGTTCTTC | MMRRC | 36715-floxF2 |
| Cxcr7 Wild Type Forward: GCTGCAAACCCGTGAACAAGG | MMRRC | 36715-wtF2 |
| Cxcr7 Common Reverse: GGGCTCTCTGGCCGTTCTCTC | MMRRC | 36175-comR2 |
| Cxcr7 Recombined Forward: TCCAGGTTGGCAGATGGATATTG | Sierro et al., 2007 | Cxcr7-P1 |
| Cxcr7 Recombined Reverse: CAGGAACCAAGGAATAGCACCC | Sierro et al., 2007 | Cxcr7-P3 |
| Cxcl12 Wild Type Forward: AAGTTGGGGACTGGGAAGAT | Ivins et al., 2015 | N/A |
| Cxcl12 Wild Type Reverse: ACTCTTGCCCAACAATCCTG | Ivins et al., 2015 | N/A |
| Cxcl12-GFP Forward: TGAACCGCATCGAGCTGAA | Ivins et al., 2015 | N/A |
| Cxcl12-GFP Reverse: TCCAGCAGGACCATGTGAT | Ivins et al., 2015 | N/A |

**Figure S1**

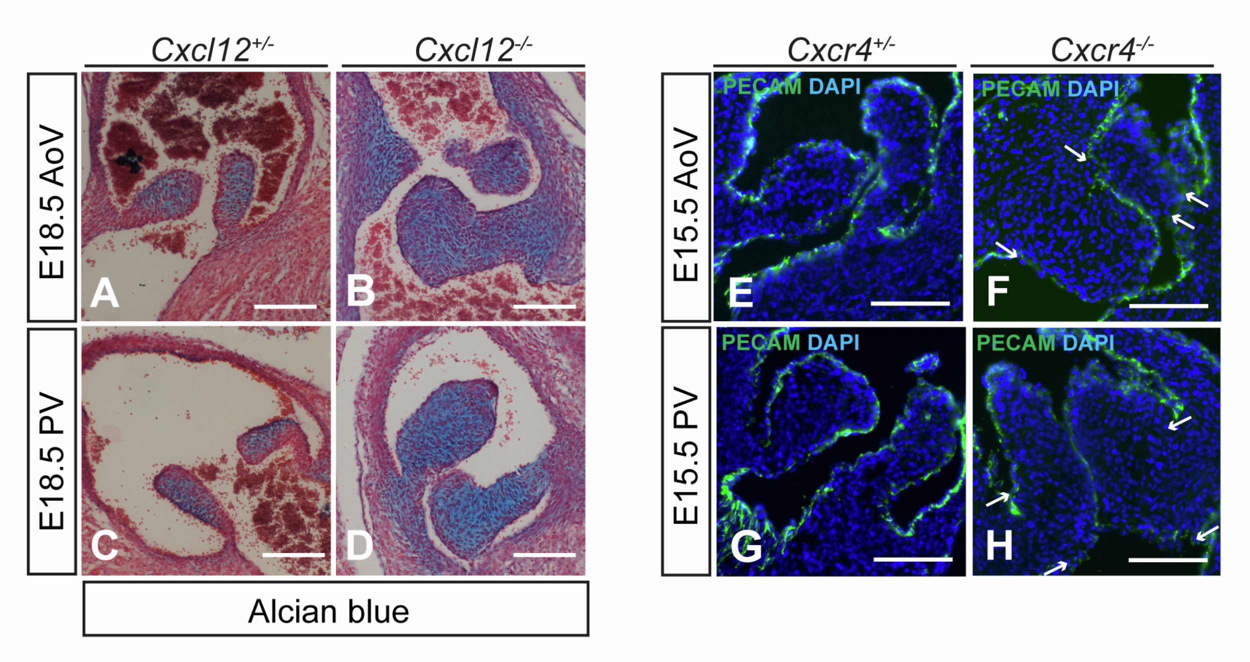


**Figure S1. Dysplasia and hyperplasia in *Cxcl12* null SLVs, related to Figure 1**

(A-D) Alcian blue staining of paraffin sections of E18.5 control (*Cxcl12*$^{+/-}$, panels A and C) and *Cxcl12*$^{-/-}$ (B and D) SLVs, showing proteoglycan deposition.

(E-H) Immunostaining of E15.5 wild type SLVs with anti-PECAM-1 antibody to show morphology of *Cxcr4*$^{-/-}$ AoV and PV (F, H) compared to controls (E, G). Nuclei were stained with DAPI (blue). Arrows in (F) and (H) indicate top and bottom edges of left and right leaflet bases; leaflet alignment is abnormal in (F) and normal in (H).

AoV, aortic valve;PV, pulmonary valve; SLV, semilunar valve. Scale bars represent 100 μm.

## Figure S2

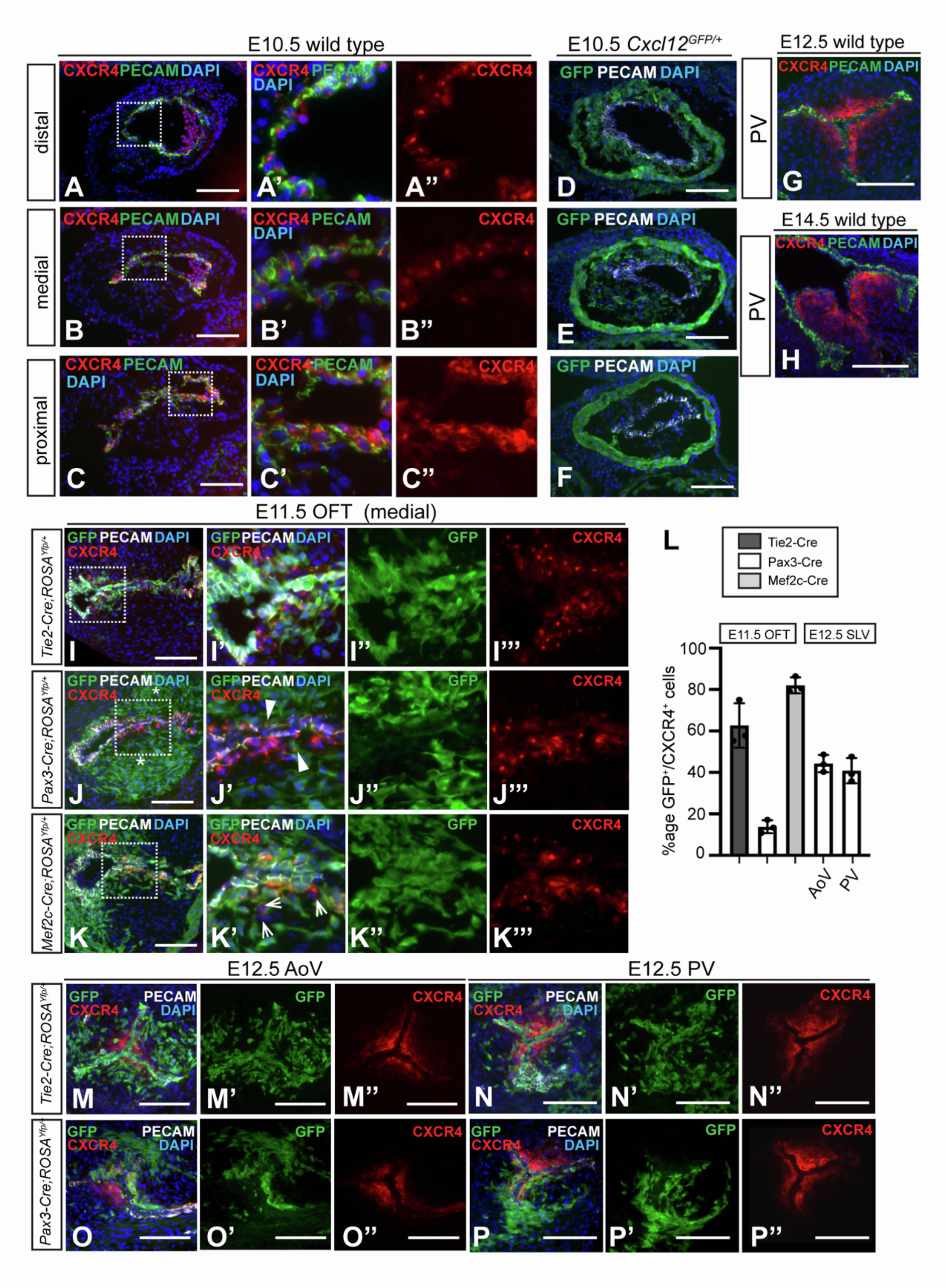


**Figure S2. Temporal and spatial variation in expression of CXCR4 and CXCL12, and origin of CXCR4$^{+}$ mesenchymal cells in the OFT/SLVs, related to Figure 2**

(A-C”) Immunostaining of E10.5 wild type OFT cushion sections to detect CXCR4 (cl. EPUMBR3) and PECAM-1 in distal (A-A”), medial (B-B”) and proximal (C-C”) OFT cushion [see (a), (b) and (c) in Figure 2A]. Nuclei were stained with DAPI (blue). Boxed areas in (A-C) are shown enlarged in neighbouring panels (A’-C’).

(D-F) E10.5 *Cxcl12*$^{GFP/+}$ OFT cushion sections were immunostained with anti-GFP antibody (and anti-PECAM-1 antibody) to show *Cxcl12* expression in distal (D), medial (E) and proximal (F) OFT cushion region [see (a), (b) and (c) in Figure 2A].

(G, H) Immunostaining of E12.5 (G) and E14.5 (H) PV sections with anti-CXCR4 and anti-PECAM-1 antibodies, as above.

(I-K”’) E11.5 medial OFT sections from *Tie2-Cre;ROSA*$^{YFP/+}$ (I-I”’), *Pax3-Cre;ROSA*$^{YFP/+}$ (J-J’”) and *Mef2c-Cre;ROSA*$^{YFP/+}$ (K-K”’) embryos, immunostained with anti-GFP, anti-CXCR4 (cl.UMB2) and anti-PECAM-1 antibodies. Boxed areas in (I), (J) and (K) are shown in the neighbouring panels. Asterisks in (J) indicate CXCR4$^-$ prongs of NC cells; arrowheads in (J’) indicate sub-endocardial CXCR4$^+$/GFP$^+$ mesenchymal cells. Arrows in (K’) indicate CXCR4$^+$/GFP$^-$ mesenchymal cells.

(L) Graph shows proportion of CXCR4$^+$/GFP$^+$/PECAM-1$^-$ (mesenchymal) cells in E11.5 OFT as a percentage of total CXCR4$^+$ mesenchymal cells in *Tie2-Cre;ROSA*$^{YFP/+}$, *Pax3-Cre;ROSA*$^{YFP/+}$, and *Mef2c-Cre;ROSA*$^{YFP/+}$ lineage-traced embryos, and in E12.5 AoV/PV (*Pax3-Cre;ROSA*$^{YFP/+}$ only). Error bars indicate mean ±SD.

(M-N”) E12.5 SLV sections from *Tie2-Cre;ROSA*$^{YFP/+}$ embryo, immunostained with anti-GFP (for lineage-positive cells), anti-CXCR4 (cl.UMB2) and anti-PECAM-1 antibodies; DAPI denotes cell nuclei.

(O-P”) E12.5 SLV sections from *Pax3-Cre;ROSA*$^{YFP/+}$embryos, immunostained as above. AoV, aortic valve; OFT, outflow tract; PV, pulmonary valve. Scale bars represent 100 µm.

## Figure S3

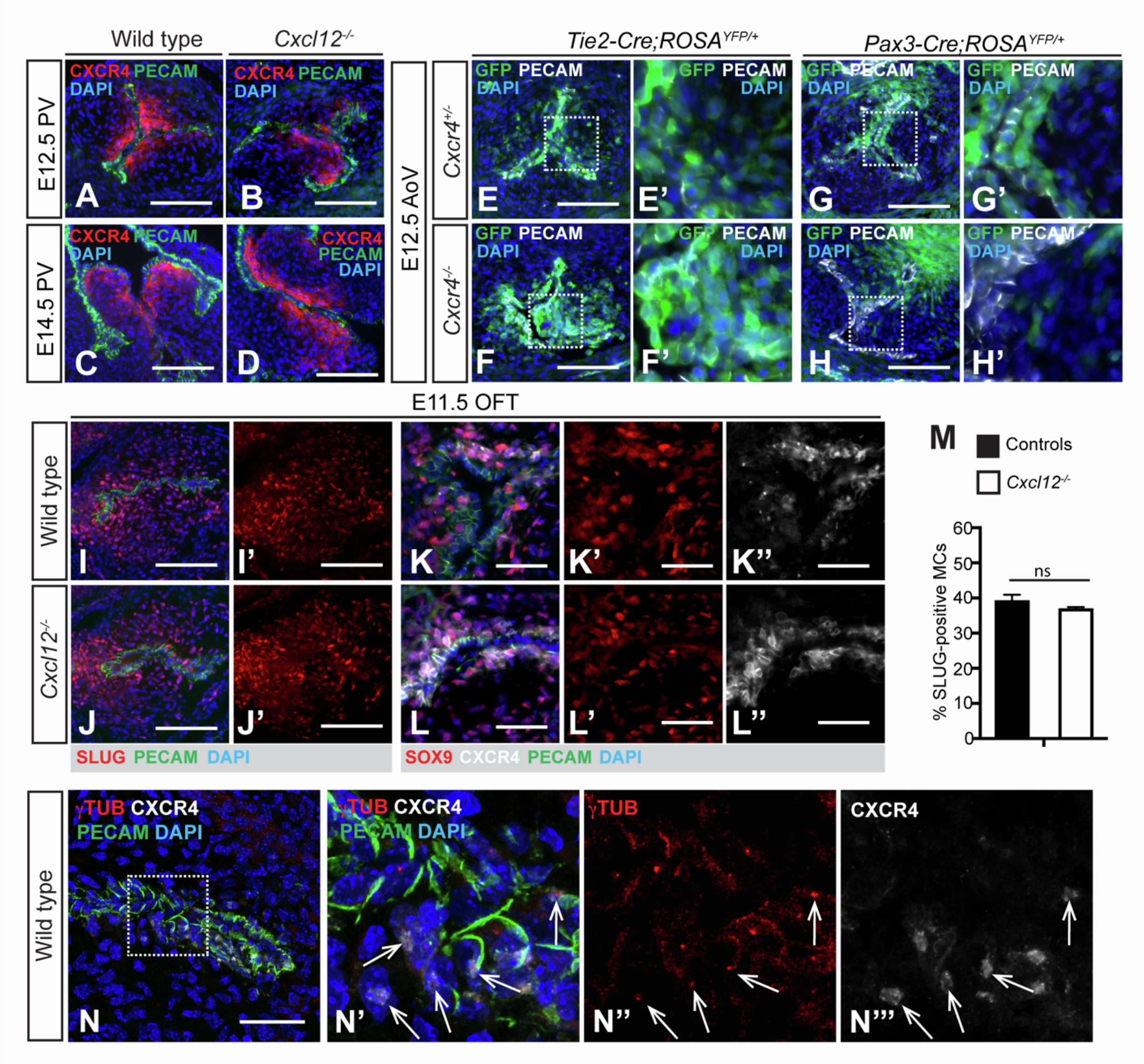


**Figure S3. Mis-localisation of CXCR4⁺ cells in *Cxcl12* nulls, but normal endo-MT, related to Figure 3**

(A-D) Defective leaflet shape and localisation of CXCR4⁺ mesenchymal cells in E12.5 (B) and E14.5 (D) *Cxcl12*$^{-/-}$ PVs compared to wild types (A) and (C). Sections were immunostained with anti-CXCR4 (cl. EPUMBR3) and anti-PECAM-1 antibodies.

(E-H') Lineage tracing in *Tie2-Cre;ROSA*$^{YFP/+}$ (E-F') and *Pax3-Cre; ROSA*$^{YFP/+}$ (G-H') E12.5 AoVs, comparing *Cxcr4*$^{+/-}$ and *Cxcr4*$^{-/-}$ sections immunostained with anti-GFP (lineage-positive cells are green), and anti-PECAM-1 antibodies. Boxed areas in (E, F, G and H) are shown in neighbouring panels (E', F', G' and H').

(I-J’) Immunostaining of wild type (I, I’) and *Cxcl12*$^{-/-}$ (J, J’) E11.5 OFT sections with anti-SLUG, and anti-PECAM-1 antibodies; nuclei were stained with DAPI.

(K-L") Immunostaining of wild type (K-K") and *Cxcl12*$^{-/-}$ (L-L") E11.5 OFT sections with anti-SOX9, anti-CXCR4 (cl. UMB2) and anti-PECAM-1 antibodies.

(M) Graph shows SLUG$^{+}$ cells as a percentage of total mesenchymal cells in *Cxcl12* controls (wild types/heterozygotes, n=3) and nulls (n=3). 5-6 sections were analysed per sample. Error bars represent ±SD, ns= non significant (Student’s t test).

(N-N'") Co-localisation of CXCR4 puncta with centrosomes (detected by anti-gamma Tubulin antibody) in wild type E11.5 OFT. Sections were co-immunostained with anti-CXCR4 (cl. UMB2) and anti-PECAM-1 antibodies. Boxed region in (N) is shown enlarged in neighbouring panels (N’-N’’’); arrows indicate double positive cells.

AoV, aortic valve; OFT, outflow tract. Scale bars represent 100 μm except for panels (K-L”) and (N) where they represent 50 μm.

## Figure S4

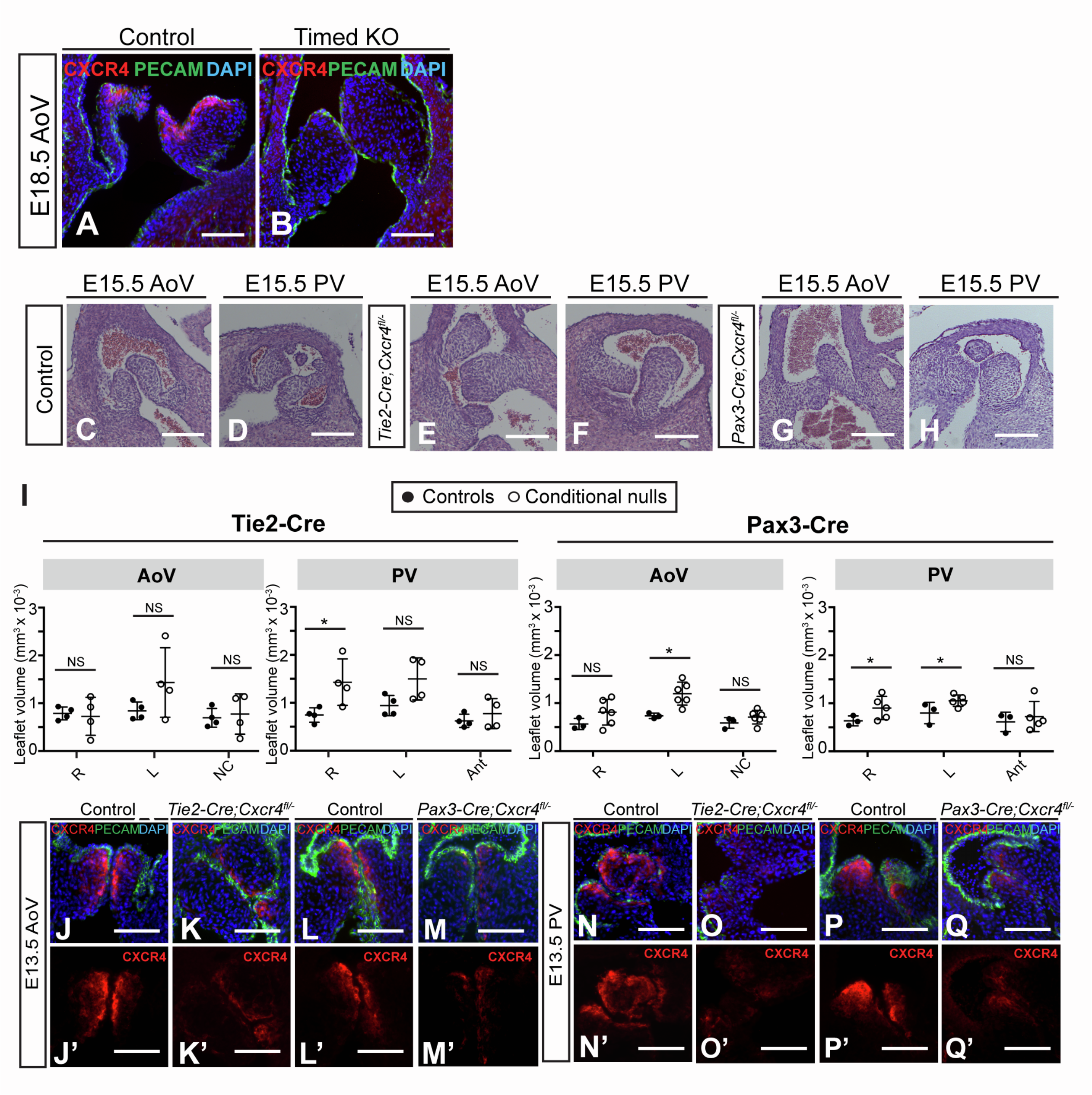


**Figure S4. CXCR4$^{+}$ cells from endocardial and neural crest lineages contribute to the developing SLVs, related to Figure 5**

(A-B) Timed knock-out of CXCR4 causes SLV hyperplasia. Hyperplasia (without misalignment) of AoV leaflets is observed in *Caggs-Cre$^{ERT}$;Cxcr4$^{fl/-}$* embryos (B) but not *Caggs-Cre $^{ERT}$;Cxcr4$^{fl/+}$* controls (A) induced with tamoxifen at E13.5. Sections were

immunostained with anti-CXCR4 (cl. UMB2) and anti-PECAM-1 antibodies; nuclei were stained with DAPI. See also Figures 5A-5E.

(C-H) H&E staining of E15.5 wild type (C, D), *Tie2-Cre;Cxcr4*$^{fl/-}$ (E, F) and *Pax3-Cre*;*Cxcr4*$^{fl/-}$ (G, H) SLVs (coronal sections).

(I) Graphs show individual SLV leaflet volumes ($mm^3$) in E15.5 *Tie2-Cre;Cxcr4*$^{fl/-}$ and *Pax3-Cre;Cxcr4*$^{fl/-}$ hearts (compared to *Cxcr4fl*$^{/-}$ littermate controls) , measured using Imaris software. Error bars represent ±SD; NS, non significant, *p<0.05 (unpaired Students t test).

(J-Q') Immunostaining of E13.5 SLVs using anti-CXCR4 (cl. EPUMBR3) and PECAM-1 antibodies, showing CXCR4 expression in *Tie2-Cre; Cxcr4*$^{fl/-}$ (K, K', O, O') and *Pax3-Cre;Cxcr4*$^{fl/-}$ (M, M', Q, Q') conditional mutants, compared to controls (*Cxcr4*$^{fl/-}$ littermate controls, J, J', L, L', N, N', P,P'). Nuclei were stained with DAPI.

Ant, anterior leaflet; AoV, aortic valve; L, left leaflet; PV, pulmonary valve; R, right leaflet; Scale bars represent 100 µm.

## Figure S5

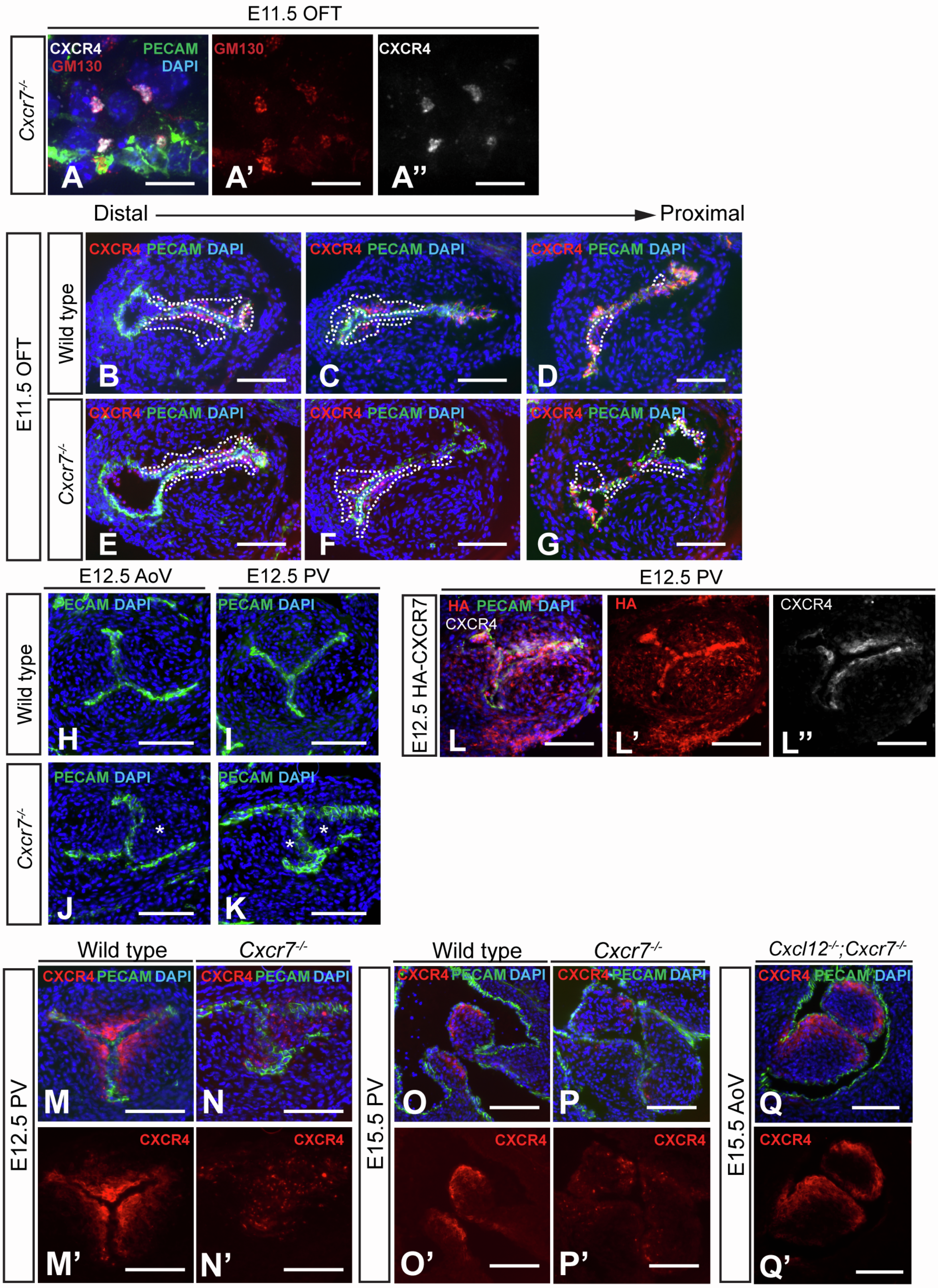


**Figure S5. Analysis of *Cxcr7* null phenotype and CXCR7 protein expression, related to Figures 6 and 7**

(A-A'') Co-localisation of CXCR4 puncta with Golgi apparatus (detected by anti-GM130 antibody) in E11.5 *Cxcr7*$^{-/-}$ OFT, co-immunostained with anti-CXCR4 (cl.EPUMBR3) and anti-PECAM-1 antibodies. Nuclei were stained with DAPI.

(B-G) Comparison of CXCR4$^{+}$ mesenchymal cell distribution in distal/medial/proximal OFT at E11.5 in wild type (A-D) and *Cxcr7*$^{-/-}$ (E-G) sections. Sections were immunostained with anti-CXCR4 (cl. EPUMBR3) and anti-PECAM-1 antibodies. Dotted lines delineate CXCR4$^{+}$ mesenchymal cells.

(H-K) Immunostaining of E12.5 wild type (H, I) and *Cxcr7*$^{-/-}$ (J, K) SLV sections with anti-PECAM-1 antibody to show valve morphology. Nuclei were stained with DAPI.

(L-L") CXCR7 expression in E12.5 PV. Sections from HA-CXCR7 mice were co-immunostained with anti-HA (detecting CXCR7), CXCR4 (cl. 2B11, detecting total CXCR4) and anti-PECAM-1 antibodies. Nuclei were stained with DAPI.

(M-P') E12.5/E15.5 wild type (M, M, O, O') and *Cxcr7*$^{-/-}$ (N, N', P, P') PV sections co-immunostained with anti-CXCR4 (cl. EPUMBR3) and anti-PECAM-1 antibodies. Nuclei were stained with DAPI.

(Q, Q') E15.5 *Cxcl12*$^{-/-}$*;Cxcr7*$^{-/-}$ AoV, immunostained as above.

AoV, aortic valve; OFT, outflow tract; PV, pulmonary valve. Scale bars represent 10 μm in panels (A-A') and 100 μm in all other panels.

## Figure S6

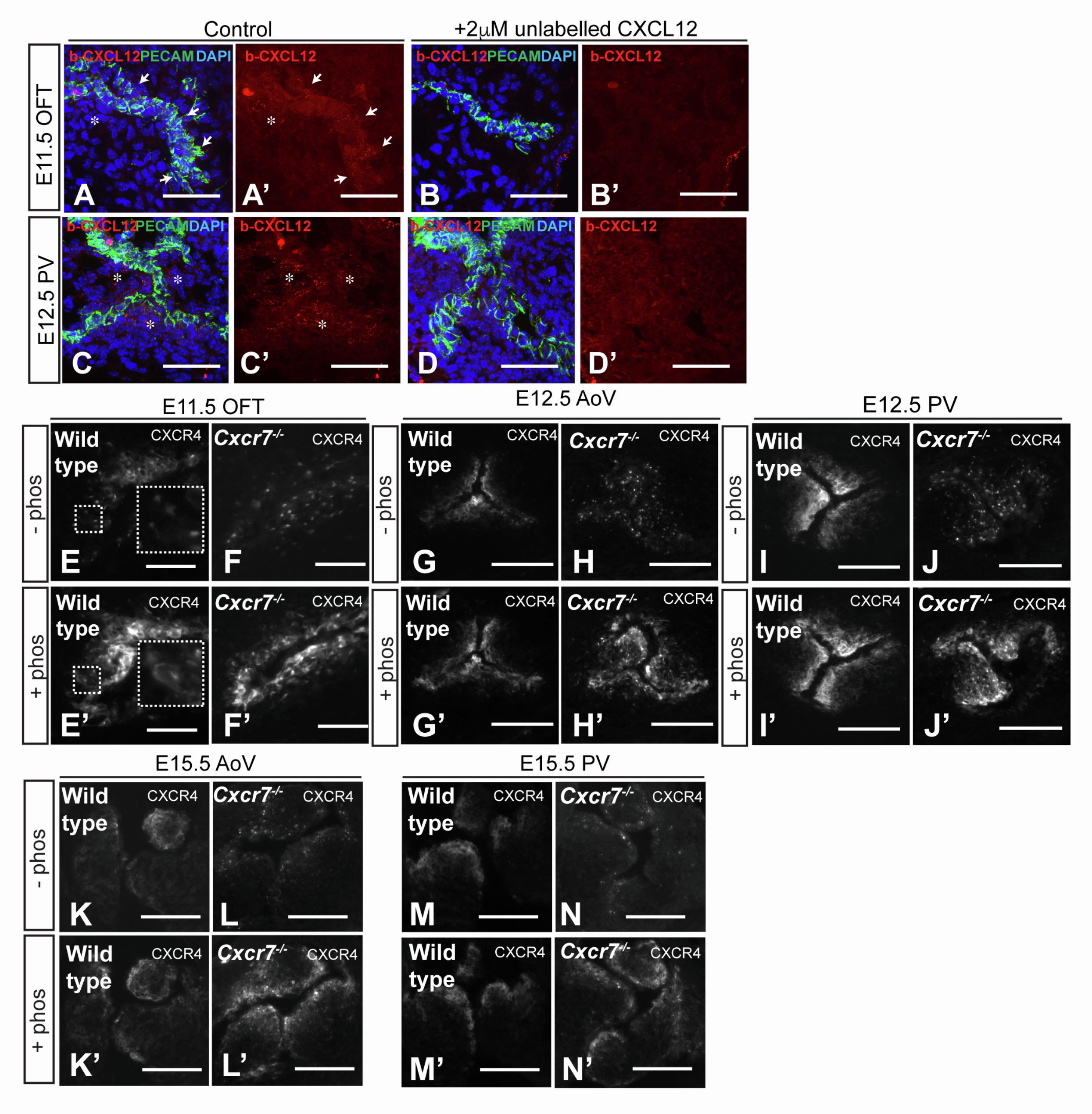


**Figure S6. SLV mesenchymal cells are over-exposed to CXCL12 in the absence of CXCR7, related to Figure 7**

(A-D') CXCL12 uptake in E11.5 OFT and E12.5 PV. Whole hearts were incubated with 200nM biotinylated-CXCL12: streptavidin-594 complex (b-CXCL12) in the presence (B-D') or absence (A-C') of unlabelled CXCL12 (x10 molar excess). Frozen tissue sections were stained with

anti-PECAM-1 antibody, nuclei were stained with DAPI. Arrows in (A) and (A') indicate signal (internalised b-CXCL12) in endocardial cells, asterisks in (A-D') show mesenchymal b-CXCL12.

(E-N') λPP treatment of wild type and *Cxcr7*$^{-/-}$ tissue sections followed by immunostaining with anti-CXCR4 antibody (cl. EPUMBR3). Serial sections from E11.5 OFT cushion (E-F'), E12.5 AoV (G-H'), and PV (I-J'), and E15.5 AoV (K-L') and PV (M-N') are shown: untreated (-phos, E-N) and treated (+ phos, E'-N'). Large dashed boxes inset in (E) and E') are enlargements of the smaller dashed boxes, showing increased CXCR4 on the plasma membrane after λPP treatment (E').

AoV, aortic valve; b-L12, biotinylated-CXCL12: streptavidin-594 complex; OFT, outflow tract; PV, pulmonary valve. Scale bars represent 100 µm, except for panels (E-F') where they represent 50 µm.

## Figure S7

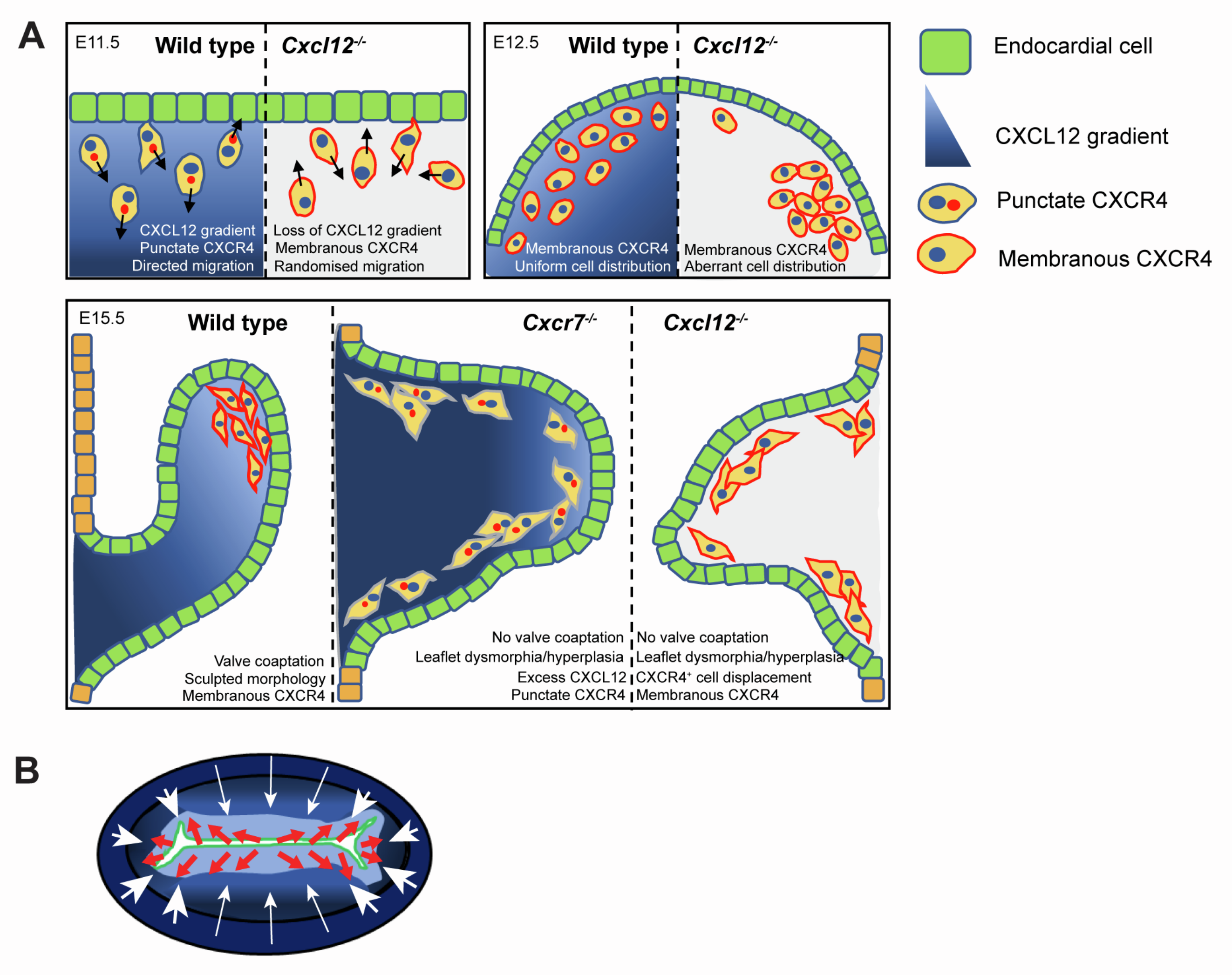


**Figure S7. Summary of *Cxcl12*$^{-/-}$ and *Cxcr7*$^{-/-}$ OFT and SLV defects, related to Figures 3, 6 and 7**

(A) Model summarising effects (intracellular localisation of CXCR4, localisation of CXCR4$^{+}$ cells, valve morphology) of knocking out *Cxcl12* and *Cxcr7* at different stages of valve formation.

(B) Schematic shows cross-section of E11.5 distal/medial OFT. Diffusion of CXCL12 (blue) is indicated by white arrows: short arrows/large arrowheads indicate lateral regions where a shorter diffusion distance potentially generates a steeper CXCL12 gradient. Red arrows show potential migratory trajectory of cells undergoing endoMT.
